# Growth of Coal Mining Operations in the Elk River Valley (Canada) Linked to Increasing Solute Transport of Se, NO_3_^–^, and SO_4_^2–^ into the Transboundary Koocanusa Reservoir (USA–Canada)

**DOI:** 10.1021/acs.est.3c05090

**Published:** 2023-11-03

**Authors:** Meryl B. Storb, Ashley M. Bussell, Sara L. Caldwell Eldridge, Robert M. Hirsch, Travis S. Schmidt

**Affiliations:** † U.S. Geological Survey WY-MT Water Science Center, 3162 Bozeman Avenue, Helena, Montana 59601, United States; ‡ Department of Land Resources and Environmental Sciences, Montana State University, Bozeman, Montana 59717, United States; § U.S. Geological Survey Water Mission Area, 12201 Sunrise Valley Drive, Reston, Virginia 20192, United States

**Keywords:** trends, load, water quality, WRTDS, selenium

## Abstract

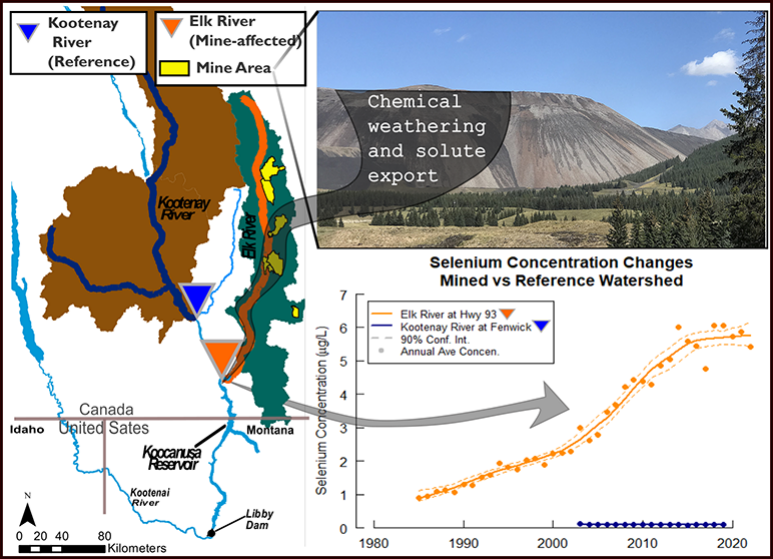

Koocanusa Reservoir (KOC) is a waterbody that spans the United States (U.S.) and Canadian border. Increasing concentrations of total selenium (Se), nitrate + nitrite (NO_3_^–^, nitrite is insignificant or not present), and sulfate (SO_4_^2–^) in KOC and downstream in the Kootenai River (Kootenay River in Canada) are tied to expanding coal mining operations in the Elk River Watershed, Canada. Using a paired watershed approach, trends in flow-normalized concentrations and loads were evaluated for Se, NO_3_^–^, and SO_4_^2–^ for the two largest tributaries, the Kootenay and Elk Rivers, Canada. Increases in concentration (SO_4_^2–^ 120%, Se 581%, NO_3_^–^ 784%) and load (SO_4_^2–^ 129%, Se 443%, NO_3_^–^ 697%) in the Elk River (1979–2022 for NO_3_^–^, 1984–2022 for Se and SO_4_^2–^) are among the largest documented increases in the primary literature, while only a small magnitude increase in SO_4_^2–^ (7.7% concentration) and decreases in Se (−10%) and NO_3_^–^ (−8.5%) were observed in the Kootenay River. Between 2009 and 2019, the Elk River contributed, on average, 29% of the combined flow, 95% of the Se, 76% of the NO_3_^–^, and 38% of the SO_4_^2–^ entering the reservoir from these two major tributaries. The largest increase in solute concentrations occurred during baseflows, indicating a change in solute transport and delivery dynamics in the Elk River Watershed, which may be attributable to altered landscapes from coal mining operations including altered groundwater flow paths and increased chemical weathering in waste rock dumps. More recently there is evidence of surface water treatment operations providing some reduction in concentrations during low flow times of year; however, these appear to have a limited effect on annual loads entering KOC. These findings imply that current mine water treatment, which is focused on surface waters, may not sufficiently reduce the influence of mine-waste-derived solutes in the Elk River to allow constituent concentrations in KOC to meet U.S. water-quality standards.

## Abstract 2

Increasing solute concentrations in the transboundary Koocanusa Reservoir are linked to coal mining in the Elk Valley, British Columbia, Canada. Historic solute loads and concentrations from paired watersheds were evaluated to identify effects to water quality and solute transport from coal mining operations.

## 1 Introduction

Worldwide, more than 260 river basins are divided and shared by multiple nations. Managing and preserving transboundary watersheds, their water resources, and cultural heritage is exceptionally difficult because political borders rarely coincide with watershed boundaries, and governments may have conflicting regulatory approaches.^1−3^ Without cooperative resource management between governments, transboundary waterways are uniquely vulnerable to the influences of human land use on water quality and ecosystem integrity. One example is large scale mining and the alteration of land surfaces and aquifers due to placement of waste rock, which are known to profoundly influence groundwater and surface water quality.^4−6^ Mining supports regional and national economies but has been shown to alter water, solute, and sediment dynamics and harm aquatic ecosystems.^7^ Understanding environmental and water-quality impacts from mines located near borders provides information that may be used to protect, restore, and manage natural resources in the complex regulatory setting of transboundary watersheds.^8^

**Figure 1:**
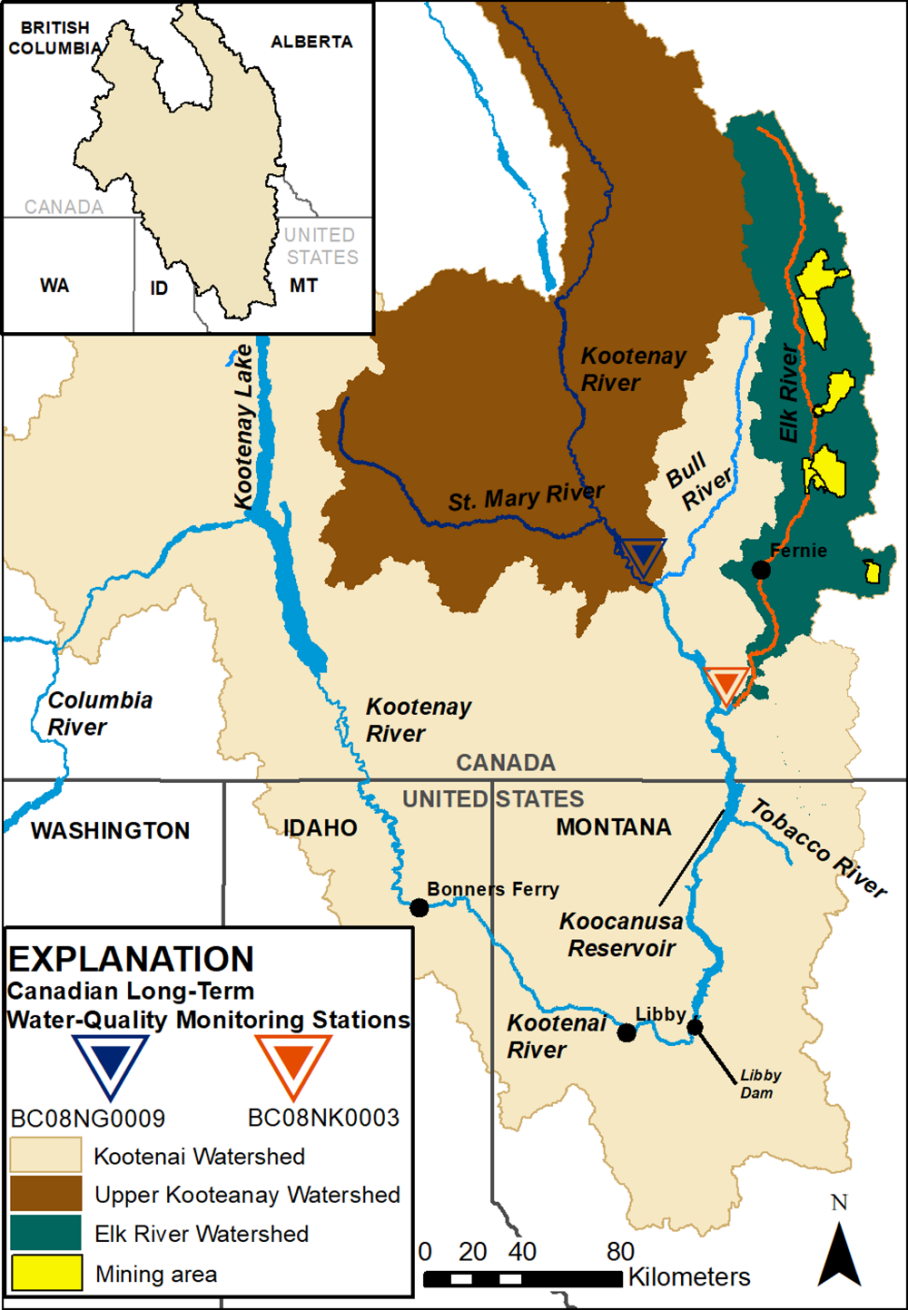
Study area map showing the Kootenai Basin (and partial Columbia River Basin) including the Elk River and Kootenay River Watersheds and other major tributaries; the Bull River, and the Tobacco River. Mine areas in the Elk River Watershed are shown in yellow, sampling locations are shown as triangles.^11,12^

Koocanusa Reservoir (KOC, also called “Lake Koocanusa”) is a transboundary reservoir that is split between northwestern Montana (MT), United States (U.S.), and southeastern British Columbia (B.C.), Canada (CA). The reservoir was impounded in 1972 by the Libby Dam, near Libby, MT (Figure 1). KOC encompasses the headwaters of the Kootenai (Kootenay in CA) River Basin. Including KOC, the Kootenai River crosses the U.S./CA border twice and drains into the Columbia River just north of where the river crosses the international border a third time (Figure 1). The Kootenai and Columbia Rivers have significant cultural importance—the watershed itself is the basis for the Ktunaxa Creation Story.^9^ Ecologically and culturally important fish resources in the Kootenai Watershed (U.S.) include the federally endangered Kootenai River White Sturgeon (*Acipenser transmontanus*), the threatened Bull Trout (*Salvelinus confluentus*), and two species of concern, Burbot (*Lota lota*) and Westslope Cutthroat Trout (*Oncorhynchus clarkii lewisi*).^10^ The headwater drainages for KOC are present on both sides of the border. However, the three largest tributaries are in B.C., and the second largest is the Elk River, which drains a watershed that contains several open-pit, coal mining operations (Figure 1).^11,12^

**Figure 2:**
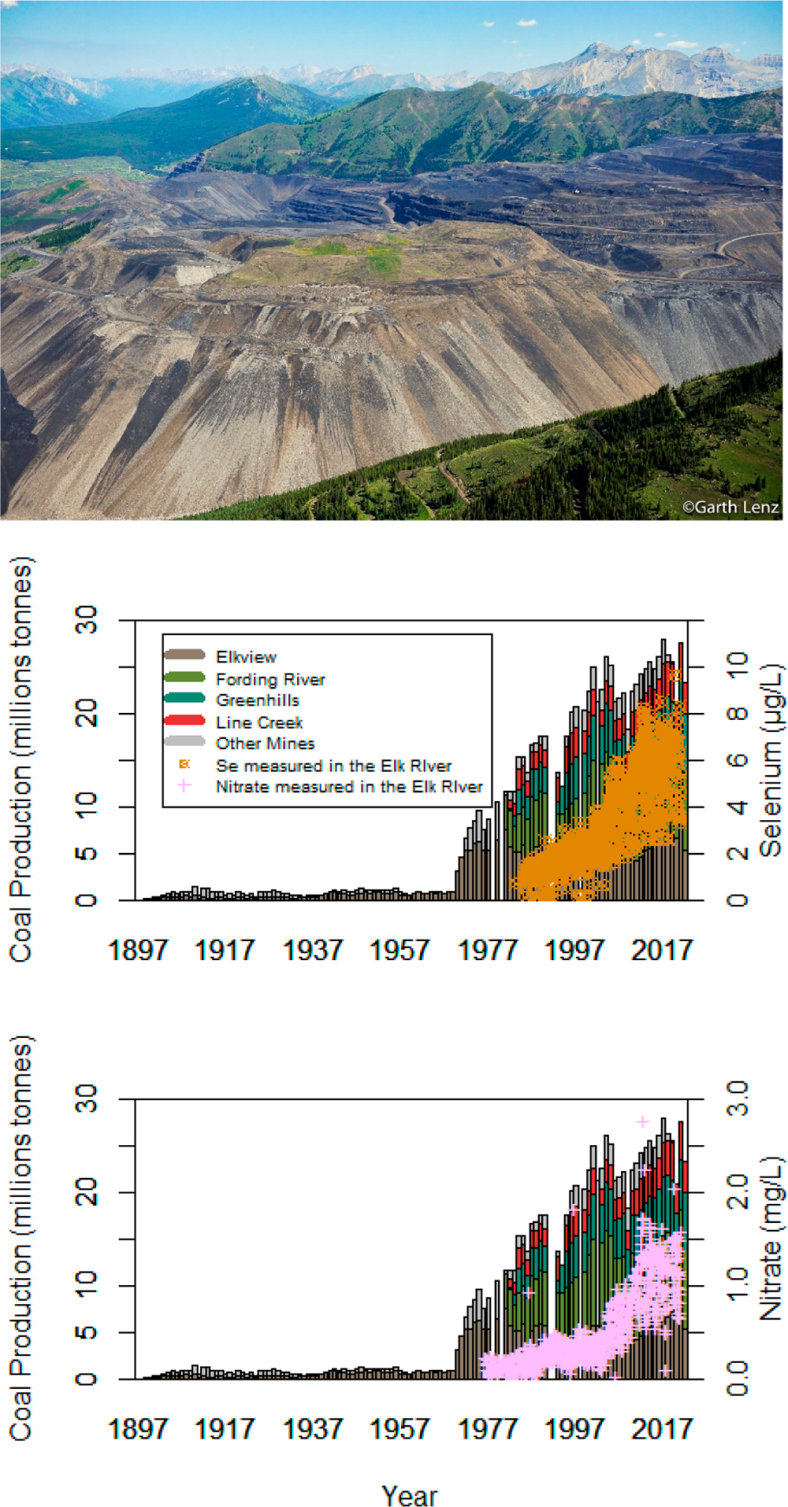
Coal production in the Elk River Watershed. Aerial image of one area of mine operations within the watershed. Copyrighted, used with permission via licensing agreement with Garth Lenz. Plots illustrate yearly coal production by mine, represented by different colors,^16^ and measured concentrations of total Se and NO_3_^–^ in the Elk River and Highway 93.^22^

Coal mines have operated in the Elk River Watershed since 1897 and are known sources of contaminants to the transboundary waters in the Kootenai River Basin (Figure 2).^5,13−15^ Current coal mines in the Elk River Watershed (Elk River Mines, ERM) are classified in Canada as open-pit mines, but they are analogous to mountain top removal coal mines in the U.S.^7^ where mining operations create abundant volumes of waste rock that are deposited in valley areas more than 100-m thick.^4,13^

Open-pit coal mining in the Elk River Watershed results in the removal of coal and waste rock and subsequent waste rock dump generation (valley fill)—these mining operations alter the slopes, physical landscape, hydrologic, hydrogeologic, and geochemical functions of the mountain headwater systems where the mines are present (Figure 2).^5,14^ As a result, these mountain headwaters have less relief and more low gradient surfaces, which alters infiltration and runoff processes.^5,14^ Geochemical processes are also altered when solid bedrock is removed and replaced with porous crushed waste rock. Waste rock dumps change the porosity and transmissivity of the system and increase the surface area and exposure of waste rock to air^16^ and water, enabling more rapid chemical weathering.^4,5,17^ Changing mining practices, including the development of new technologies, treatments, and best managements also further complicate the landscape and runoff and infiltration mechanisms, including lag times that affect solute movement through waste rock.^18−21^

**Figure 3:**
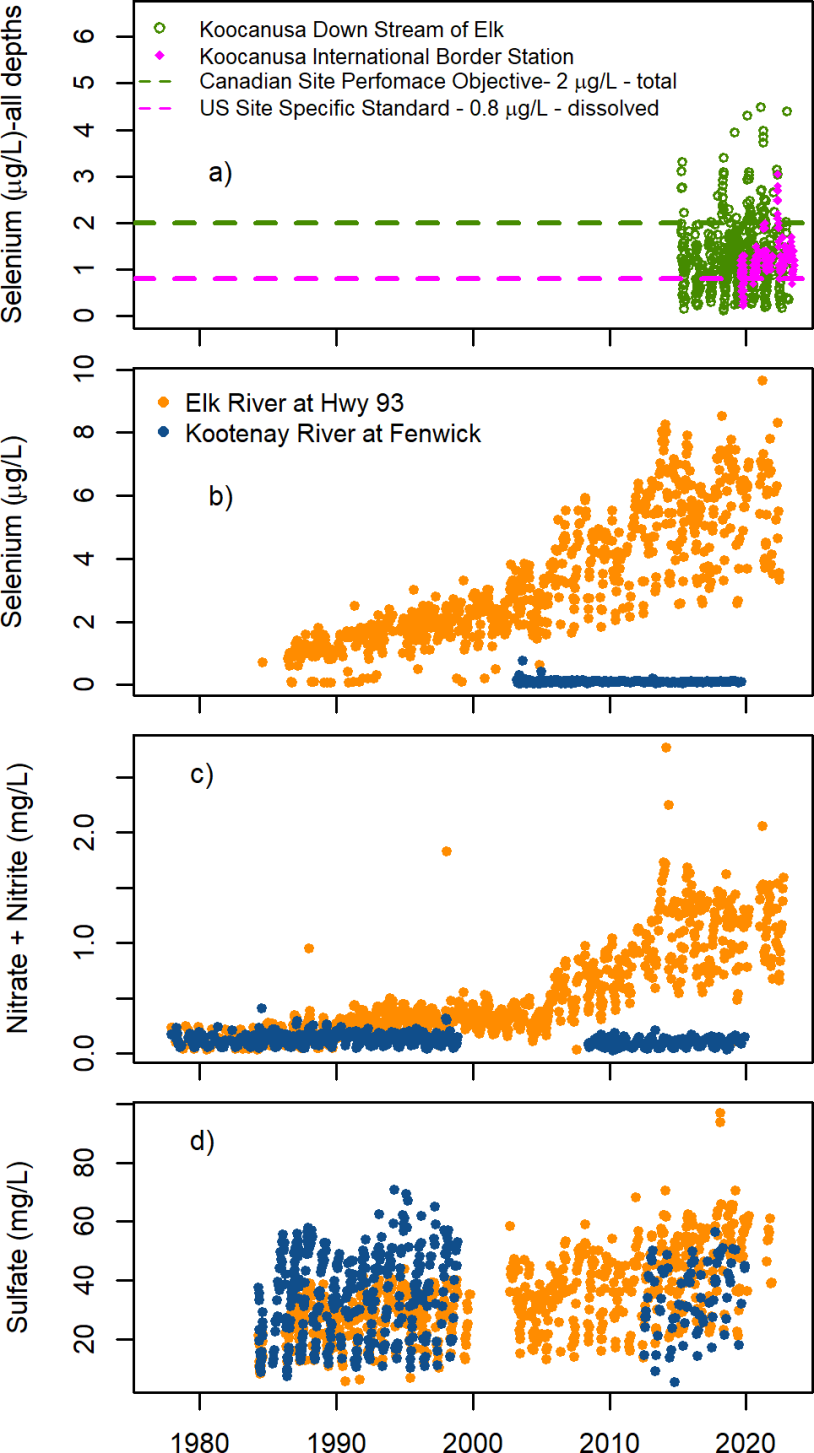
Solute concentration measurements in Koocanusa Reservoir, the Elk River at Highway 93 and the Kooteanay River at Fenwick.^22^ (a) Koocanusa Reservoir total Se concentrations measurements at a Canadian compliance point (Lake Koocanusa South of Elk River, green)^46^ and dissolved Se on the United States side of the international boarder (U.S. Geological Survey Site ID 12300110, magenta)^24^ with their respective regulatory criteria.^45,46^ Concentration measurements of total Se (b), NO_3_^–^ (c), and SO_4_^2–^ (d) at the Elk River at Highway 93 (orange) and the Kootenay River at Fenwick (blue).^22^

Past literature has correlated Se and NO_3_^–^ concentrations in the Elk River with the volume of waste rock produced in the ERMs (Figure 2); however, the mechanisms driving the changing concentrations (e.g., the increase in concentrations around 2005) are not well understood.^5,13^ Likewise, Se, NO_3_^–^, and SO_4_^2–^ concentrations measured in the Elk River where it enters KOC have been increasing since concentrations were first measured in 1984, and concentrations are also increasing in KOC (station no. 12300110) (Figure 3).^22−24^ Water-quality sampling on the Elk River was initiated roughly 80 years after coal mining began, but it is likely that mining affected water quality before monitoring started.^15^

Coal-associated waste rock contains sulfide minerals (pyrite, most commonly) and organosulfide compounds which are associated with Se and other trace elements.^25−27^ Se is of particular importance because its crustal concentrations can be enriched up to 82 times in coal-bearing geologic formations.^27^ Chemical weathering through the oxidation of sulfide minerals releases SO_4_^2–^ and associated trace elements to both surface water and groundwater.^28^ Increasing NO_3_^–^ concentrations in the Elk River have been tied to blasting practices from coal mining operations,^13^ and excess ammonium may originate directly from coal.^29^ Release of these mine-waste-derived solutes has negative implications for beneficial uses, including the potential to harm ecosystems in downstream water bodies.

KOC is oligotrophic due to low background phosphorus concentrations and limited sediment delivery to the downstream section of the reservoir because of the Libby Dam. Increasing NO_3_^–^ loads from the Elk River coupled with limited phosphorus has the potential to alter food webs through an imbalance of nutrients.^30−33^ Food web effects from the nutrient imbalance have known ecological consequences downstream of the reservoir in the Kootenai River in MT and Idaho (ID), where phosphorus is now being added to improve food webs for fisheries.^30,34^

Se is an essential trace element for life, but excess Se can be toxic to vertebrates and invertebrates.^35^ In egg-laying organisms, Se substitutes for sulfur in proteins causing teratogenesis (deformities) in early life stages, among other effects.^31,36−39^ Thus, elevated Se entering from the Elk River poses a risk to organisms and the ecological function of KOC and the entire Kootenai Watershed.

In 2012, KOC and the Kootenai River were listed as impaired for Se on Montana’s Clean Water Act section 303(d) list.^40^ Idaho’s listing for Se occurred in 2020 with a second section of the Kootenai listed in 2022.^41,42^ From 2015 to 2020, the State of MT and province of B.C. worked to develop site-specific Se standards to protect water resources in KOC. The water column Se criterion of 0.8 ug/L (dissolved Se) was set for the U.S. portion of KOC (along with fish tissue criteria) (Figure 3) and 3.1 μg/L (dissolved Se) for the Kootenai River in MT and ID.^43−45^ However, as of September 20, 2023, B.C. has yet to officially establish a site-specific water-quality guideline for the CA portion of the reservoir. B.C. is the primary regulatory entity, and they have specified water-quality performance objectives (i.e., CA regulatory criteria for water column concentrations) at order stations (i.e., CA regulatory compliance points) associated with mining permits, which are site-specific objectives that differ from CA water-quality guidelines.^46^ The water-quality performance objectives for Se, at the order station within the CA portion of KOC, match the provincial recommended guideline value of 2 μg/L (total) (Figure 3a).^47^ Water-quality criteria for Se are now being regularly exceeded on both sides of the border. At the time of publication, water Se concentrations collected at the international boundary (USGS Site ID 12300110) have not been less than the U.S. site-specific regulatory standard since July, 2020 (Figure 3a).^24^

Here, we aimed to gain a better understanding of how the history of ERM operations has influenced the quality of waters flowing into the U.S. via a retrospective analysis of Canadian water-quality and discharge records. Our analysis included three constituents (Se, NO_3_^–^, and SO_4_^2–^) in the two largest tributaries of KOC (The Kootenay River and Elk River), near their entries to the reservoir. For all three constituents in each river, we built empirical models of concentration, using weighted regression as a function of time trend, discharge, and season (WRTDS).^48^ Based on these models, we estimated daily and annual concentrations and loads and evaluated trends in flow-normalized concentrations and loads. Through a paired watershed study with three objectives, our goal was to address the following research question: How does the export of potential contaminants such as Se, NO_3_^–^, and SO_4_^2–^ reflect the history of coal mining land use and waste rock management in watersheds? Our first objective was to model and estimate the masses of Se, NO_3_^–^, and SO_4_^2–^ that enter KOC annually from the two major tributaries and assess how the annual delivery of solutes has changed through time via flow-normalized trends in concentration and load. Our second objective was to determine the relative contributions of Se, NO_3_^–^, and SO_4_^2–^ over time, from the two tributaries into KOC. Lastly, we looked for evidence of changing solute dynamics in the two tributaries in relation to the coal mining operational history, including the recent implementation of water treatment at the ERM.

While public concern and scientific study related to mining contaminants in the Elk River have been previously documented in government reports, Canadian and U.S. national news sources, and primary literature,^5,14,27,49−52^ this study provides the first comprehensive and comparative analysis in the primary literature of historical water-quality effects to U.S. waters from solutes derived from coal mine operations in the Elk River Valley. Our efforts demonstrate how flexible analysis techniques (WRTDS) can be used to improve our understanding of changing solute dynamics and the fate and transport of mine-waste-derived solutes (Se, NO_3_^–^, and SO_4_^–2^).

## 2 Methods

### 2.1 Site Information

KOC is 145 km long and is bisected by the international border in southeastern B.C., CA, and northwestern MT, U.S. (Figure 1).^53^ There are four gauged tributaries that contribute water to KOC; the three largest are Canadian tributaries, the Kootenay, Elk, and Bull Rivers—which together supply approximately 87% of the inflow to the reservoir, with roughly 50% coming from the Kootenay River and 25% from the Elk River (Figure 1).^23,54,55^

Large growth of mining operations in the Elk River Watershed occurred in the 1970s, with the transition from underground mining to large scale surface mining with valley-fill waste rock dumps (Figure 2).^16,56^ In 2020, ERM was responsible for producing 80% of Canada’s annual steelmaking coal exports and 43% of B.C.’s mining revenues. In 2021, ERM produced 24.6 million tons of coal, with the majority exported to the Asia Pacific region.^57,58^ In 2022, the ERM encompassed over 145 km^2^ (3%) of the Elk River Watershed (SI Figure 1and SI Table 1a,b). At present (2023), there are also three additional proposed mines and one proposed mine expansion.^56^ As of 2020 the ERM had generated over 6.74 billion bank (*in situ*) cubic meters (B.B.C.M) of waste rock,^29^ a 43% increase from the 4.7 B.B.C.M of waste rock that was present in 2010.^59^ Current permitted mine operations allow 11.03 B.B.C.M of waste rock, so volumes could nearly double from their present sizes under existing mine permits (SI Table 2).^29^ The areal extent of waste rock dumps is specified by permitting,^5,60^ so future waste rock generation from existing operations is likely to alter the geometry of the dump areas by increasing their depth.

In 2013, based on rising concentrations of five constituents (Se, NO_3_^–^, SO_4_^2–^, cadmium and calcite) in the Elk River, B.C. began requiring ERM to address mining contamination.^61^ Remedial measures implemented by ERM included piloting of water treatment technologies to remove Se and NO_3_^–^ beginning in late 2014, leading to 4 operating treatment facilities by the end of 2022 (SI Table 4).^29^

### 2.2 Data Compilation

Water-quality and discharge data were obtained from the online B.C. Water Tool (downloaded on 7/7/2022)^55^ and the Environment and Climate Change Canada (ECCC) data portal (downloaded on 11/12/2022).^62^ Water-quality data for the Kootenay River were obtained from the Kootenay River near the Fenwick site (B.C.08NG0009), which has a drainage area of 11,754 km^2^. Discharge and water-quality sampling locations were not coincident, so discharge data from the Kootenay River at Fort Steele (station ID 08NG065)^55^ were adjusted based on the drainage area ratio between the discharge monitoring and the water-quality sampling locations (SI Figure 2).^22^ The Elk River is 220 km long and has a drainage area of 4,450 km^2^ to the water-quality monitoring site located at Highway 93 Near Elko (B.C.08NK0003).^23^ Because the discharge and water sampling locations were not coincident, the Maintenance of Variance Extension, Type 2 (MOVE.2)^63^ for record extension was used to extend the daily discharge record at the Elk River at Phillips Bridge site (08NK005),^55^ which is the closest gauge, but discharge measurements were discontinued after 1996. Record extension for Phillips Bridge was based on the discharge relationship with the Elk River at the Fernie site (08NK002) (correlation coefficient 0.9805) (SI Figure 2).^63,64^ A drainage area ratio correction was then applied to adjust for the differences in contributing area between the Phillips Bridge discharge records and the Highway 93 water-quality monitoring location.^22^ Additional information is in the SI Methods, including additional site information and geology. Concentration and discharge input files, in addition to the models, associated metadata, and additional details are available in ref (22).

Both the Elk and Kootenay Rivers have snowmelt-dominated flow regimes, generally characterized by peak discharges in late May through early June, transitioning to baseflow recession in the late summer and early fall, and finally steady low discharge dominated by baseflow contributions throughout the winter months (SI Figure 3).^5^ Water-quality samples at each location were generally collected two times each month over the period of record for each solute and generally spanned the range of flow conditions (SI Figure 4). Additional information regarding data quality screening, and corrections, are available in ref (22).

### 2.3 Analysis Methods

Weighted Regression on Time, Discharge and Season (WRTDS) was implemented using R Studio (version 1.4.1106), and the EGRET (version 3.0.7), and EGRETci (Version 2.0.4) packages.^48,65,66^ Six individual models were generated, with one model for each constituent of interest at each site. Additional details, including the governing equation are included in the Supporting Information (SI Analysis Methods).

#### 2.3.1 Evaluation of Flow Stationarity

To determine the appropriate implementations of WRTDS (stationary flow-normalization or generalized flow-normalization), eight metrics of variation in annual stream discharge (SI Figures 5 and 6) were examined for monotonic trends at each river location.^67^ The statistics were evaluated using the nonparametric Mann-Kendall trend test with Theil–Sen slope (SI Figures 5 and 6). In addition, Quantile-Kendall plots were examined for changes in discharge quantiles for the periods of record with discrete water-quality data (Elk 1979–2021; Kootenay 1979–2019) (SI Figure 7).^68^ These statistical tests suggested that trends in discharge were small and not statistically significant; therefore, we implemented WRTDS using stationary flow-normalization.

#### 2.3.2 Flow-Normalized Trends

Trends in the flow-normalized concentration and load were examined visually and quantitatively for each model using the WRTDS framework. WRTDS was developed to evaluate the combined effects of discharge, season, and interannual trends in water quality. It does this by creating a statistical model of concentrations for each day in the record as a function of discharge, trends in time (expressed in decimal years), and season (SI Analysis Methods; Kalman). The model results were integrated over the frequency distribution of the discharge to compute flow-normalized estimates. These estimates are designed to allow a comparison of year-to-year variations in concentration and load that are independent of the effects of variations in discharge. Removing this source of variation facilitates the identification and estimation of long-term, nonmonotonic trends.^48,69^

The EGRETci package was used to estimate the uncertainty associated with these trends through block bootstrapping.^70^ This method constructs confidence intervals around a trend by randomly subsampling the data set and recreating the WRTDS model over many iterations to capture variability in the trend estimate. Uncertainty was described using likelihood terminology following Hirsch, Archfield and De Cicco,^70^ where a trend likelihood is described based on the percentage of increasing or decreasing trends from hundreds of bootstrapped iterations, with higher likelihoods corresponding to higher percentages.^70^

Trend estimates for load are generally presented in the Results and Discussion section as yield (i.e., area normalized load). Loads are presented as yields to make results more directly comparable because the Kootenay Watershed has more than twice the contributing area of the Elk Watershed (Figure 1).

Additionally, we chose to explore general effects that the recent implementation of ERM water treatment (post 2015) may have had on the contaminant loading trends at the Elk River at the Highway 93 sampling location (80–120 km downstream of mining operations). Daily mass removal data from ERM water treatment for Se and NO_3_^–^ through September 2022 are available in ref (22). We carried out a mass balance calculation for Se using the combined daily Se mass removals from all treatment locations. The calculations make two simplifying assumptions. The first is that the Se removed by the treatment plants would have otherwise been transported conservatively (i.e., in its entirety) 80–120 km downstream to the Highway 93 sampling location. The second assumption is that the load reduction at the monitoring location on any given day is equal to the running mean of Se removal for the prior 30 days. This averaging was selected to account for different treatment locations upstream and to account for surface water advection and dispersion as well as exchange of the solute between the surface water and groundwater systems. This second assumption results in a smoothing of the overall treatment effect assessment but does not have a significant effect on the concentration reduction estimates at longer time scales (e.g., seasonal to annual). The smoothed data were used to construct a concentration surface using the same indexing (for time and logQ) as the original Se concentrations computed from the monitoring data within the Elk Se WRTDS model.^22^

#### 2.3.3 Load Estimates

The EGRET package^65^ was used to provide daily estimates of concentration and load, which were then summarized into a time series of annual mean concentration and annual load using a state space modeling approach with the WRTDS_Kalman method.^71,72^ The WRTDS_Kalman method uses the WRTDS model and the measured sample values to compute optimal estimates for each day in a manner that accounts for serial correlation in the model residuals. It is a method that has been shown to generate some of the most accurate estimates of load based on comparisons with measurements.^71,72^ Comparisons between observed and modeled daily loads and WRTDS and WRTDS_Kalman annual estimates are in SI Figures 8 and 9.

#### 2.3.4 Concentration–Discharge Relationships

Concentration–discharge relationships (C–Q) were plotted and assessed for variation with respect to seasons and over time, providing a summary of solute dynamics and insights into changing watershed flushing and dilution processes. WRTDS captures changes in C–Q relationships over time and generates a modeled 3D surface that shows expected concentrations across a range of possible discharge values for each day in the period of record (SI Figure 10). Information contained within this 3D surface allows for inferences about the effects of changing hydrologic conditions, evidenced through changing behavior in the C–Q relationship over time.^48,73,74^

Two different visualizations were explored. The first set of visualizations are like traditional C–Q plots, but on an arithmetic scale, and relationships at three different days over time are shown on the same graph. Multiple iterations of these graphs were examined looking at historical changes during high and low discharge times of year. The second set of plots looked at changes in concentration over time for each constituent at specific discharges based off a flow duration curve generated for 60 days surrounding a specific date (high, 95th quantile; intermediate, 50th quantile; and low discharges, 5th quantile), during peak discharge (June 13) and low discharge (January 1) times of the year. These two graphical tools provide a visual assessment of patterns that can illustrate potential changes in solute delivery dynamics over time because patterns in the relationship of C–Q provide perspective on the mechanisms related to the mixture of precipitation water and stored water in streamflow generation.

There are three common patterns that are frequently described for C–Q relationships: dilution, mobilization, and chemostasis. Often these patterns vary between individual solutes and watersheds, so they are useful for characterizing the average behavior of a watershed.^75−80^ As such, a change in C–Q behavior over time may indicate hydrologic shifts, such as changes in land use that affect basin characteristics, changes or transformations in constituents and sources, or altered flow paths (i.e., solute delivery dynamics).^76,81,82^

## 3 Results and Discussion

### 3.1 Concentration

Within the two watersheds, both land use and bedrock geologies are generally different, suggesting the possibility that the background concentrations of solutes might vary (SI Methods, Geology). Background Se concentrations have the potential to be higher in the Elk River than the Kootenay River, based on bedrock geologic sources independent of mining.^83^ The mean Se concentration on the Kootenay River (2003–2019) is near 0.1 μg/L and is likely to have decreased 10% over this time (Table 1). The annual mean concentration for Se on the Elk River at Highway 93 in 2022 was 5.77 μg/L, but in 1985 it was 0.89 μg/L (Table 1).^22^ The 1985 value is 295% greater than concentrations on the Elk River upstream of the mines and in the neighboring Flathead River watershed, based on values that were measured by Hauer and Sexton^15^ (0.3 μg/L). There is limited literature surrounding background concentrations in the Elk River, but values upstream of the mines and the concentration increases early in the record (SI Table 3) suggest that concentrations of mine-related contaminants may have been increasing since before concentrations were initially measured.

**Table 1:**
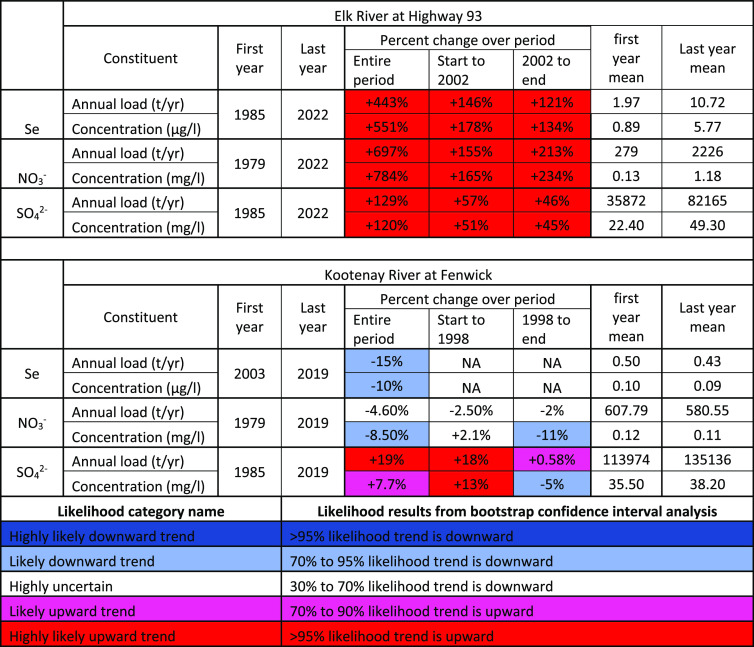
Trends in Loads and Concentrations (Flow-Normalized) For Select Time Periods for the Elk River at Highway 93 Bridge and the Kootenay River at Fenwick^22^^,^^a^

a Uncertainty is described in terms of the likelihood from bootstrapped replicates.

Like Se, NO_3_^–^ and SO_4_^2–^ concentrations in the Elk River have followed a trajectory where average, maximum, and minimum concentrations are all increasing, in addition to increases in amplitude in annual patterns among high and low concentrations (Figure 3b,c,d). NO_3_^–^ concentrations have remained stable or decreased marginally in the Kootenay River (Figure 3b,c,d).^84^ NO_3_^–^ and SO_4_^2–^ concentrations entering KOC do not exceed current CA regulatory guidance for water quality.^85,86^ However, NO_3_^–^ concentrations in the Elk River have increased 784% between 1979 and 2022, more than any other solute (Table 1). NO_3_^–^ concentrations in the Elk River were like those in the Kootenay River when concentrations were first measured in the 1980s, but recently measured high values each year are nearing half the 3 mg/L CA guidance level (Table 1).^85^

Since the year 2000, measured Se concentrations in the Elk River have constantly exceeded recommended ambient water-quality guidelines to protect aquatic life set by both the Canadian Council for Ministers of the Environment (1 μg/L) and the British Columbia Ministry of Environment and Climate Change Strategy (alert concentration: 1 μg/L; guideline: 2 μg/L).^47,87^ In KOC, dissolved Se concentrations have exceeded U.S. regulatory criteria since July, 2020 (Figure 3a).^45^ In contrast, Se concentrations in the Kootenay remain 1–2 orders of magnitude lower and stable (Figure 3b).

The Elk River location at Highway 93 is not a CA regulatory compliance point; therefore, concentrations here are being compared to the CA recommended federal and provincial Se guidelines (total).^47,87^ In 2021, the average annual measured Se concentration in the Elk River where it enters KOC was 5.87 μg/L, the maximum was 9.65 μg/L, and the minimum was 3.51 μg/L. We used the WRTDS model output to calculate the expected number of days that modeled concentrations exceeded the guideline criteria (SI Methods, Exceedance Probability). For the federal guideline of 1 μg/L, exceedances increased from 70 days a year in 1984, to 252 in 1990, to 350 in 2000, and by 2006 exceedances were greater than the federal/provinical alert guideline for more than 360 days per year, for each year until the present. Similarly, for the 2 μg/L provincial guideline, the expected number of exceedances per year rose from 7 days in 1984, to 31 days in 1990, to 217 days in 2000, and by 2010 it is greater than the CA recommended guidance concentration for more than 360 days per year for every year since 2010.

### 3.2 Loads

Loads discussed in this section are based on estimates optimized for accuracy (WTRDS_Kalman) and not flow-normalized.^71,72^ Within the Elk and the Kootenay Watersheds, snowmelt runoff is the time of year when most of the discharge moves through each system and with it most of the mass of solutes is delivered to KOC over the water year. The mean annual discharge for the Kootenay River is roughly twice that of the Elk River (SI Figure 3 and SI Table 3), and generally, the proportion of water from the two tributaries discharged into KOC did not change over the last 40 years (SI Table 3). On average, the Elk River contributed 29% of the combined discharges, but in the past decade contributed 95.3% of the Se, 76% of the NO_3_^–^ and 38% of the SO_4_^2–^ to KOC from the two tributaries combined (SI Table 3). Annual variations in loads for both rivers were driven by year-to-year variations in discharge, and within the Elk River variation in loads were also coupled with the large increase in solute concentrations from mining operations.

The masses of SO_4_^2–^ delivered to KOC from these rivers were the most similar relative to the other solutes (SI Table 3). Mining in the Elk River Watershed has added additional mass to background weathering and other SO_4_^2–^ sources, which was evident by the increase in annual load estimates over time, from 25% of the combined load in the late 1980s to 38% in the 2010s (SI Table 3).

NO_3_^–^ load increased more than the other two constituents within the Elk River over its respective period of record. This increase is likely driven by ammonium nitrate used in mine blasting^13^ and to some extent geogenic ammonium ions in the coal-bearing strata.^29^ In 1979, when NO_3_^–^ was first measured, roughly 30% of the NO_3_^–^ entering KOC was coming from the Elk River, a proportion like the discharge volume (concentrations were similar in both rivers), but by 2014, it had increased up to 83% of the combined NO_3_^–^ contributed to KOC (SI Table 3).

Annual loads of Se have grown by over 1 order of magnitude over the past 35 years (SI Table 3). When water-quality monitoring was initiated in the Elk River in 1984, estimated loads were already 3–4 times greater than loads entering the KOC from the Kootenay River (SI Table 3).

The mass of NO_3_^–^ and Se being delivered to KOC is now dominated by contributions from the Elk River, despite its much lower discharge volume (SI Table 3). Discharge in the vicinity of waste rock dumps in the Elk River Watershed has been shown to be attenuating, with decreasing peak and stormflows and increasing baseflows.^58^ Thus, the timing of solute mass delivery may shift toward a greater proportion during baseflow periods, due to increased transient storage and solute sources from growing volumes of waste rock.^4^ Likewise, several studies have shown that transport is the limitation for solute delivery out of waste rock within the Elk River Watershed.^5,13,14^ This limitation is present despite increased infiltration capacities that can be three times greater than the infiltration capacity of the natural catchment.^60^ This suggests that if more flow through waste rock occurred, more solute mass would be mobilized—an important consideration given changing precipitation patterns and form (rain vs snow) as a result of climate change.

A portion of the most contaminated surface waters are now being treated, and the volume of water to be treated is planned to increase through 2027.^88^ In 2015^14^ and 2018,^5^ Wellen, Shatilla and Carey found that mining practices, including surface reclamation, the shape of waste rock piles, and the age of waste rock dumps, also have the potential to reduce Se delivery to water bodies, suggesting operational practices, which change in time, are likely to influence loads (increase or decrease). Water treatments may reduce loads to KOC going forward, but the magnitude and timing of that reduction are not well understood. Likewise, there has been little published study of surface water–groundwater interaction in mine-affected areas and areas downstream. A recent update to the Regional Water Quality Model (RWQM) added a surface water–groundwater partitioning component, suggesting solute movement in groundwater is an important mechanism that needs to be understood in the context of downstream water quality.^29,89,90^ Loads from both shallow and deep groundwater are a source of uncertainty in this system, and very little is known about the effects on deep groundwater from waste rock dumps on top of bedrock limestone with high karst potential.^89,91^ Presently only surface water is treated, and current plans are to treat only surface waters into the future.^29^ With limited knowledge surrounding surface water–groundwater interaction and potential groundwater contamination, it is unclear if treatment of surface water alone will sufficiently reduce the mass of solutes moving downgradient in the watershed and into KOC to meet U.S. water-quality regulations (Figure 3a).

### 3.3 C–Q Relationships

For each of the three solutes in the Kootenay River, the C–Q relationships for individual solutes were consistent over time, suggesting consistency in the solute delivery mechanisms for the watershed (SI Figure 11a–c). Se and SO_4_^2–^ both showed a dilution signal. Se shows a slight decrease across all concentrations from 2003 to 2019, while SO_4_^2–^ shows an increase across all discharges between 1986 and 2019, but more so at lower discharges (SI Figure 11a,c). NO_3_^–^ exhibited a mobilization pattern, suggesting that the mechanisms behind the Kootenay’s delivery of NO_3_^–^ to KOC is driven by runoff solute delivery mechanisms, coupled with an overall decrease across all concentrations between 1986 and 2019 (SI Figure 11b).

**Figure 4:**
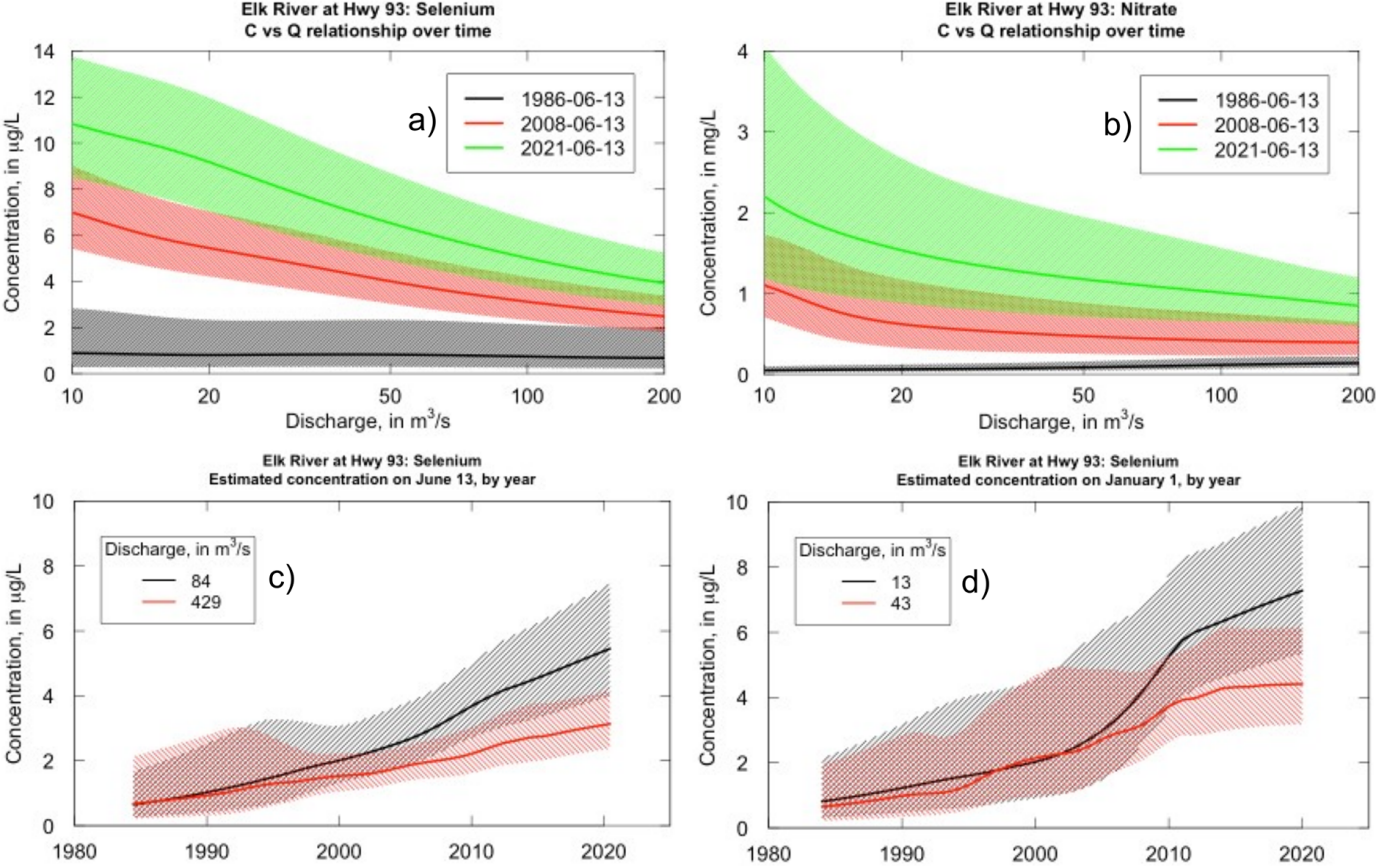
Concentration vs discharge relationships for total Se (a) and NO_3_^–^ (b) in the Elk River at Highway 93.^22,65^ Colored lines (a,b) show modeled C–Q relationships for three different dates, illustrating changes in the solute behavior over time. Shading (a, b) shows the 90% prediction interval for each date. (c,d) illustrates changes in concentration over time at two different discharges and two times of the year. (c) illustrates patterns at the high discharge time of year (June 13) and (d) shows a low discharge time of year (Jan 1). Lines (c, d) represent the 5th (black) and 95th (red) percentiles for flow duration curves for 60 days around each date (30 days before and after). Shading (c, d) shows the 90% prediction interval for each flow. Additional solutes and Kootenay River data are shown in SI Figures 11 and 12.

In contrast to the Kootenay River, the Elk River C–Q relationships for Se and NO_3_^–^ changed with time (Figure 4), while SO_4_^2–^ maintained a consistent dilution signal, but the magnitude of concentrations increased across all discharges (SI Figure 11f). In 1986 Se and NO_3_^–^ in the Elk River exhibited C–Q relationships that were indicative of mobilization or chemostasis solute delivery mechanisms (Figure 4). However, by 2008 and into 2021, the solute delivery for both Se and NO_3_^–^ changed to dilution (Figure 4a,b). Like SO_4_^2–^, as time progressed both Se and NO_3_^–^ exhibited an overall increase in concentrations across all discharges, with the largest magnitude of increase occurring at the lowest flows.

In addition to more traditional perspectives on C–Q relationships, we used information within the WRTDS models to evaluate how concentrations have changed at different discharges (i.e., seasonal high, average, or low discharge) during different seasons (Figure 4c,d and SI Figure 12).^65^ In the Elk River for all three constituents, we observed that solute concentrations at lower discharges increased faster than those at higher discharges (SI Figure 12). This was the case during both high discharge (June 13) and low discharge (January 1) times of the year (Figure 4c,d). However, the magnitude of concentration increase was greatest during the lowest discharges, at the baseflow time of the year (Figure 4c,d). Patterns shown in Figure 4 display a notable change in relative rates of increase (slope) that occurred in the mid-2000s, where the concentrations began to increase more rapidly across the different discharges—this is particularly evident in Se during the low flow time of year (Figure 4d). In contrast, patterns across discharges in the Kootenay River for all three solutes during high and low discharge periods are relatively flat over time (not shown). The shift in Se and NO_3_^–^ C–Q signals (Figure 4a,b), as well as the largest concentration increase during low flows (Figure 4), suggests that processes controlling solute delivery to surface water have changed over the last 40 years in the Elk River, with a notable increase in the lowest flows relative to higher flows taking place in the early to mid-2000s (Figure 4d).

Concentrations of solutes at baseflow conditions are generally a good indicator of the chemistry of shallow groundwater; existing reports suggest that most streams in the Elk Valley gain shallow groundwater.^58^ Therefore, shifts in C–Q behavior (increased concentrations during baseflow) may reflect increased solute loadings to streams from shallow groundwater. However, increasing concentrations at low discharges are also consistent with observations made by Nippgen et al.,^4^ who suggest waste rock dumps may be altering the hydrologic storage and release of solutes within Appalachian coal mine affected watersheds, causing increased solute mobilization during the baseflow period via surface water drainage from dumps. The switch in Se and NO_3_^–^ C–Q behavior to dilution and the largest increase in concentrations at the lowest baseflows in Figures 3 and 4 suggest that the ERM may now behave like a point source, with a near continuous release of higher concentration solutes that are being diluted by water from unmined portions of the Elk River Watershed. The changing solute behavior in the Elk River could be the result of contaminated groundwater downgradient of waste rock dumps and or increases in surface water sources (concentration and/or discharge) emerging from waste rock dumps—suggesting that additional investigation to understand the mechanisms driving the change in solute delivery in the Elk River Watershed may support understanding the source(s). In contrast, solute dynamics in the Kootenay River have remained relatively consistent over time.

### 3.4 Trends

#### 3.4.1 Differing Trends from the Paired Watersheds

**Figure 5:**
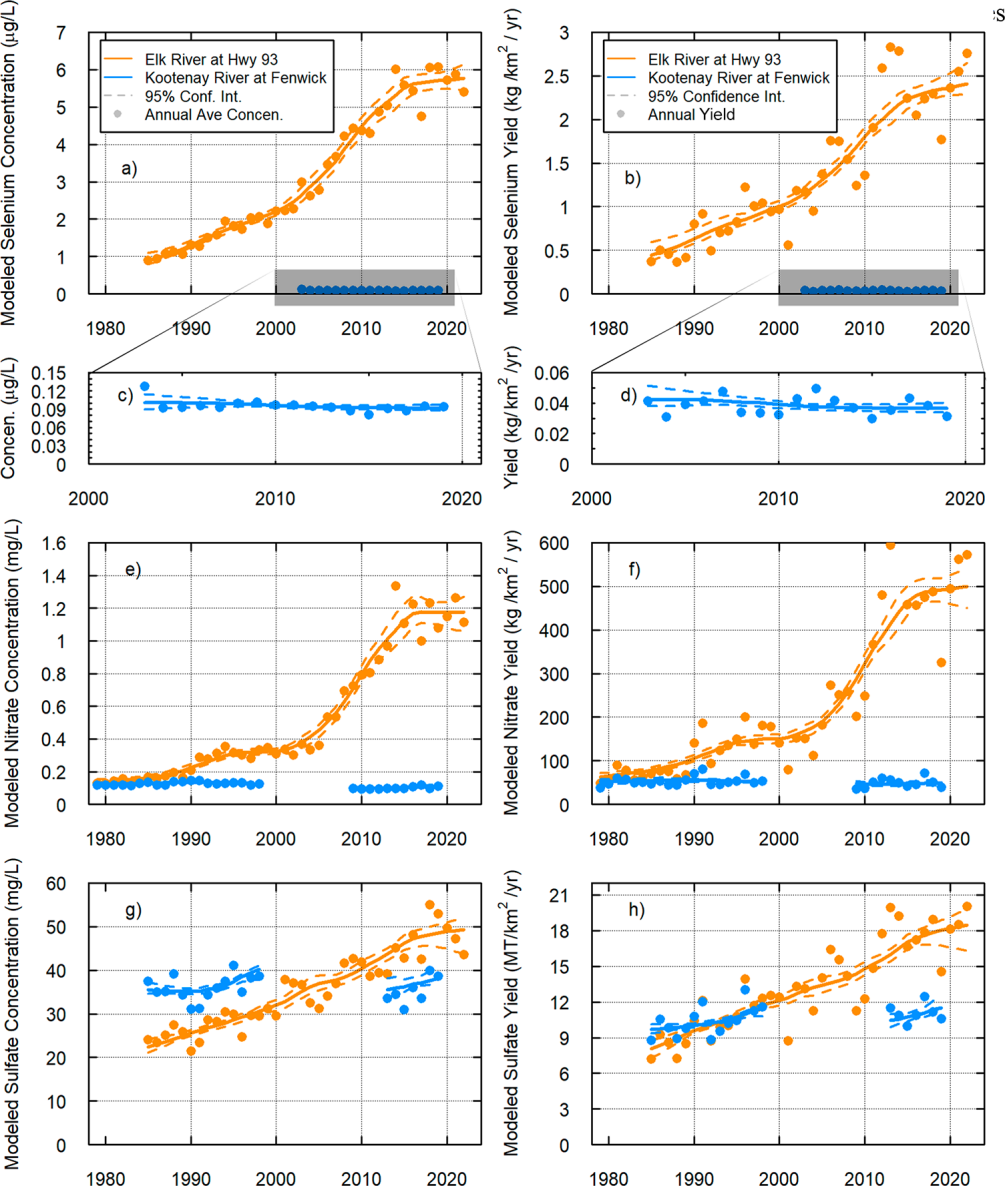
Flow-normalized trends for the Elk River (orange) and the Kootenay River (blue).^22,48,65^ Concentration trends for each constituent are on the left (a, c, e, g), and trends in yield (i.e., area normalized load) are on the right (b, d, f, g).^22^ Each row is a constituent: (a, b) total Se, (c, d) total Se for the Kootenay River only, (e, f) NO_3_^–^, and (g, h) SO_4_^2–^. Solid dots are mean annual concentration or annual yield. Dashed lines are 90% confidence intervals.^65^

Trends in load are presented in this section as yields, to facilitate direct comparisons between the two watersheds, which are different in size. In the Kootenay River there are gaps in the SO_4_^2–^ and NO_3_^–^ record and a shorter Se record due to discrepancies in concentration measurements after methods changes; thus, the time periods for comparison vary slightly (Figure 5). However, the flow-normalized trends (concentration and yield) for these constituents in the Kootenay River either decreased (Se and NO_3_^–^) or increased marginally (SO_4_^2–^) (Figure 5 and Table 1).^22^

The flow-normalized trends of Se, NO_3_^–^, and SO_4_^2–^ for both concentrations and yields in the Elk River document significant water-quality changes (Figure 5 and Table 1); Se concentrations in the mine-affected tributaries to the Elk River are among the largest in published literature, and the increases in Se and NO_3_^–^ in the Elk River at Highway 93 are the largest percent increases documented in the primary literature that are known to the authors.^35,92^ Flow-normalized concentrations of Se, NO_3_^–^, and SO_4_^2–^ in the Elk River increased over their respective periods of record by 551% (Se), 784% (NO_3_^–^), and 120% (SO_4_^2–^). Trends in yield mirrored those in concentration but were slightly smaller in magnitude for Se (443%) and NO_3_^–^ (697%) and slightly larger for SO_4_^2–^ (129%) (Table 1). This occurred while marginal decreasing trends in Se and NO_3_^–^ and a slight increase in SO_4_^2–^ were observed in the Kootenay River.

Modeled annual average NO_3_^–^ concentrations in the Kootenay River and Elk River were nearly the same when monitoring began in 1984 (Figure 5c). Since 1984, concentrations of NO_3_^–^ have been declining in the Kootenay. There were likely significant declines after the closure of a fertilizer manufacturing plant (ammonia phosphate) on the Saint Mary River in 1987 (a major tributary to the Kootenay, Figure 1).^93^ Conversely, NO_3_^–^ concentrations have been on an upward trajectory in the Elk River from mining operations.

SO_4_^2–^ concentrations in the Elk River in 1984 were lower than concentrations in the Kootenay River; concentrations were comparable between the two rivers in the mid 2000s but have been higher in the Elk River ever since (Figure 5e). Generally, SO_4_^2–^ and Se weather from the same parent material; however, the difference in concentration trends over time (Figure 5a, g) suggests that different geochemical or biogeochemical mechanisms affect these solutes in different ways within the Elk River Watershed.

In the Kootenay River, percent change for SO_4_^2–^ and Se yields is greater than the respective changes in concentrations. However, NO_3_^–^ is the opposite, again suggesting that mechanisms delivering NO_3_^–^ are different from the other two solutes (Table 1 and Figure 5), which aligns with the mobilization signal that was observed in the C–Q relationship for NO_3_^–^ on the Kootenay River (SI Figure 11). Overall concentration and yields entering KOC from the Kootenay River have been consistent historically, especially in comparison to the large magnitude increase in all three solutes from the Elk River.

For NO_3_^–^ and Se in the Elk River, the increase in concentration has been larger in percentage terms than the corresponding increase in yield, but this pattern was the opposite for SO_4_^2–^ (Table 1 and Figure 5a–d). This is driven by the larger relative increases in concentration during baseflow, but due to low discharge volumes during those times, trends in load have not increased at the same rate. The relative magnitude of the increase in SO_4_^2–^ (concentration and yield) was less than the other two constituents, and the trend was more consistent in terms of the magnitude of the slope through time (Figure 5e, f). The difference in the SO_4_^2–^ trends in the Elk River (compared to NO_3_^–^ and Se) may have been driven by chemical weathering of other geologic sources of sulfate, delivering higher baseline concentrations (SI Methods, Geology).

#### 3.4.2 Mining Operations Drive Trends in the Elk River

Three distinct time periods of increasing trends in the NO_3_^–^ and Se concentrations were observed in the Elk River (Figure 5a–d). The first period was from the start of the record through the early 2000s and was characterized by a moderate (relative) slope. The second period showed an increase in the slope around 2002, suggesting the concentrations and corresponding solute delivery were increasing at a faster rate. The third period coincided with a taper in the slope around 2015. The 2015 inflection is noteworthy for NO_3_^–^ and Se as the first reduction in the slope of the trend that has been underway for 3 to 4 decades. What caused the taper in both the concentration and yield trends around 2015 is uncertain, but we pose three hypotheses that could explain changes in hydrologic processes that could cause a slowing in the rate of increase late in the record:

(1) The RWQM^58^ incorporates lag times to account for the time it takes for weathering or release of solutes after waste rock deposition to begin appearing in surface waters. Those times in the RWQM average to 7 years across all watersheds. Similar work in the primary literature has also shown that 8 year lag times were necessary to model solute delivery within the West Line Creek mine site. Past work has tied concentration with waste rock volume and areal extent.^5,13,14^ Other work has also shown the possibility of some depletion to occur in this time scale for NO_3_^–^; however, it is unlikely that depletion of Se is occurring on the same time scale. The similarity in trends between Se and NO_3_^–^ suggests that the pattern in trends is not driven by depletion alone.^27,94,95^ Thus, we hypothesize that the lag times coupled with decreases in waste rock production from economic conditions^16^ could explain the inflection in trend near 2015, as they began to present themselves roughly 7 years after the economic recession of 2008 when there was a decrease in coal production (Figure 2). This hypothesis could be tested by evaluating annual waste rock production over time; however, those data are not publicly available since 2010.^96^

(2) Alternatively, other mine engineering processes could have affected the trend. A transition to deeper waste rock dumps with fixed aerial extent could increase flow path lengths and limit or delay solute transport temporarily.^5,13,25^ Past work has shown that solute loss in waste rock dumps is driven by vertical percolation and not lateral inputs of water.^5^ Similarly, Villeneuve et al.^60^ describe three stages in the evolution of the dump footprint for the West Line Creek operation, which is part of the Line Creek Mine in the ERM: first a linear increase in dump area from 1981 to 1998, followed by decreasing rates of areal expansion between 1998 and 2003, and a relatively constant dump area from 2003 to 2014, suggesting an increase in thickness (longer vertical flow paths) during this time.^5^ Here we hypothesize that the inflection point could occur because of the lagged effect of these longer flow paths through waste rock, resulting in longer time periods before solutes will enter downstream surface waters. However, longer flow paths are also associated with more oxidation of sulfide minerals and may cause a subsequent increase in concentrations and loads later in time.^5,13,25^

(3) The third hypothesis is that the reduction in the trend slope is a result of water treatment. ERM have invested in active and passive water treatment technologies to mitigate NO_3_^–^ and Se concentrations into the Elk River Watershed; pilot efforts began as early as 2014, but the first treatment system was not fully operational until 2018 (SI Table 4).^29^ To explore the effect that the treatment may have had on the contaminant loading at the Elk River at the Highway 93 Bridge sampling location (80–120 km downstream of mining operations), we carried out a mass balance calculation (described here in terms of Se) using daily mass removals from ERM water treatment facilities between October 2015 and September 2022.^22^

**Figure 6:**
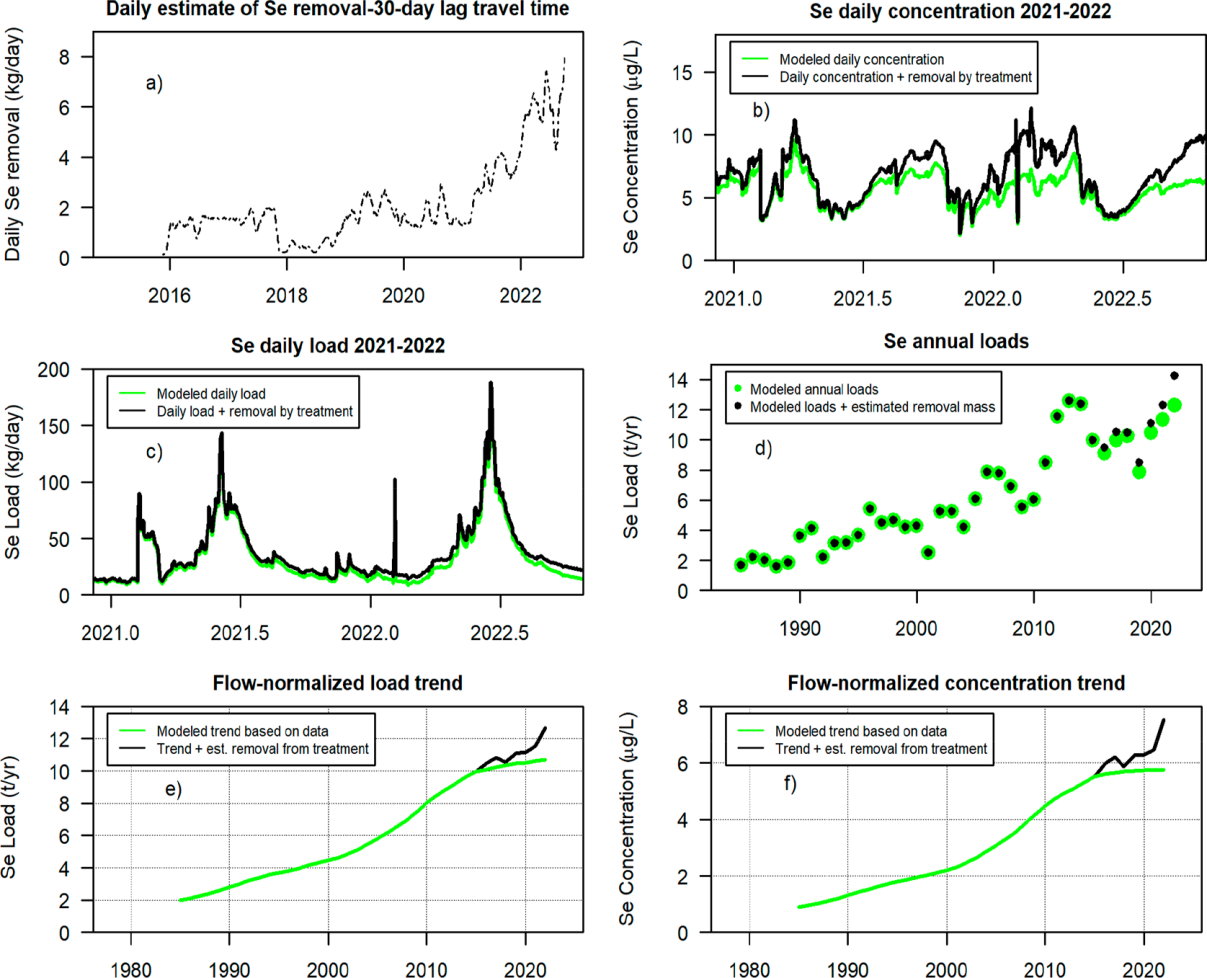
Modeled influence of Se mass removal from three mine water treatment facilities in the Elk River Watershed.^22^ Estimated Se removal by water treatment in the Elk River at Highway 93, using a 30 day lagged running mean (a). Estimated daily concentration (b) and load (c) of Se in the Elk River at Highway 93 (only 2021 and 2022 are shown for temporal resolution). The green line (b,c,e,f) is an estimate based on the model optimized for accuracy (WRTDS_Kalman).^71,72^ The black line (b,c,e,f) is based on this same model with the addition of the estimated amount of Se that was removed by treatment. Green dots (d) are the estimated annual load of Se for the Elk River WRTDS_Kalman model. Black dots (d) are the annual loads if there was no treatment. The flow-normalized trends for load (e) and concentration (f) in black, are an estimate of the trends in the absence of treatment, compared to what was observed (modeled) in green.

Figure 6a shows the sum of the 30 day running mean of the daily Se removal from all treatment locations. Figure 6b shows modeled daily concentrations at Elk River and the estimated concentrations that could have occurred if there were no treatment removals upstream, assuming our assumptions are reasonable. There were notable reductions in simulated concentration during times of low discharge (late summer through early spring). But, at times of high discharge (June), the decreases due to treatment were limited. Figure 6d shows a load perspective; the simulated effect of the treatment plants approaches a 40% reduction for portions of the months of lowest discharge in 2022 but is limited in the months of the highest discharge. For high discharge months, the amounts removed are minor (less than 5%) compared to the total amount of Se being transported by the Elk River (SI Figure 13a). Additional perspective on the effect of treatment can be seen in SI Figure 13b,c (like the patterns in Figure 4); concentrations are generally higher in the months of lower discharges. Concentration increases between 2006 and 2015 and 2016–2022 were substantial for high and low discharge segments of the year. The effect of the treatment was greater for the months of low discharge than it was for the months of high discharge.

In general, with our assumptions in mind, our analysis suggests that water treatment has been successful in reducing concentrations during the months of lower discharge (when concentrations tend to be highest). But, for the high discharge months, the effect of treatment has been modest. This translates directly to treatment effects on loads, suggesting that treatment will have less effect on annual loads and more on some seasonal and annual average concentrations. Accurate understanding of treatment effects on concentrations downstream may be supported by a more complete understanding of the hydrologic and hydrogeologic system between the ERM and the outlet to KOC than is currently available. How mass removals by treatment actually affected concentrations and loads at the Highway 93 site (without our two simplifying assumptions) is not yet clear.

Incorporating masses removed from treatment back into the WRTDS model of Se in the Elk River provides support for a combination of hypotheses. The plateau in the Se trends is still present even with mass removals incorporated (i.e., without treatment), and we see an increase in the delivery rate after 2021 (Figure 6e,f). This suggests that the reduction in rate around 2016 may not only be the result of treatment but may be more likely attributed to hypothesis 1, showing a possible lagged rebound in production postrecession, or hypothesis 2, showing how dump construction practices that are focused on increasing height of dumps have resulted in longer flow paths and further lagged times for solutes to enter downstream surface waters. The absence of the rise in solute concentration and load around 2021 (Figures 5a,b and 6e,f) suggests that in recent years concentrations could have resumed a more rapid rate of increase without treatment, which aligns with increases in solutes from longer flow paths in hypothesis 2. The modeled trends (Figure 5a–d) and trends incorporating removal (Figure 6e,f) suggest that the overall patterns in trends may be more likely attributed to hypotheses 2 and 3, with the plateau driven by operational practices that increased flow path lengths and coincident timing that would have resulted in another rapid increase in the trend around 2021 if treatment had not been present at its current capacity. Fundamentally the combination of changes in waste rock production, waste rock management, and water treatment are likely to drive the magnitude of downstream solute delivery.

## 4 Implications and Future Outlook

Through a retrospective analysis of water-quality and discharge data, this study indicates that increasing mine waste derived solutes have been exported from ERM operations to the transboundary KOC, while solute delivery from the Kootenay River has remained largely unchanged. The large differences in load contributions from these two watersheds point to the potential further increase in solutes into the Elk River with coal mine expansions or new coal mines and for solutes to continue to be released after the end of mining.^20,95^ The physiographic setting of the ERM and their operational practices have resulted in tons of Se and thousands of tons of other mine wastes (NO_3_^–^, SO_4_^2–^) entering U.S. waters (SI Table 3) that are now resulting in exceedances of U.S. and CA water-quality regulations in KOC (Figure 3a).^22,24,45,46,97^

Within the mining affected watershed of the Elk River, beginning in the early 1980s, there have been large, documented increases in both load and concentration for Se and NO_3_^–^.^5,14,22,23^ There have been large relative increases in the concentration of SO_4_^2–^ as well. In the case of Se, the Elk River is now delivering on average 95% of the combined annual Se mass to KOC. Large increases and recent plateaus in the concentration and load trends suggest that changes may be driven by waste rock production, waste rock dump geometry (vertical vs lateral expansion), and mine surface water treatment in the last 1–2 years; these processes seemingly dictate the magnitude of downstream delivery of solutes.

The changes in the C–Q relationships and increasing baseflow concentrations in the Elk River indicate increased chemical weathering of solutes due to increased waste rock volume and year-round mobilization of solutes into surface water. Solutes may be transported to the Elk River via waste rock dump discharge to surface water and/or through groundwater discharge to surface water. Changes in the solute delivery dynamics in the Elk River suggest that the hydrologic processes responsible for delivering solutes have changed over the past four decades. In the 1980s C–Q relationships for Se and NO_3_^–^ showed patterns indicative of surface runoff mechanisms exhibited through mobilization and chemostatic behavior but are now dominated by dilution. This is further evidenced by the largest increase in concentrations of all three solutes in the Elk River during the lowest discharges during the late fall and winter baseflow months. However, a better understanding of surface water–groundwater interaction may support observations in the study by filling a knowledge gap as the increase in solute concentrations at low discharges could be partially attributed to a contaminated groundwater source.

Past studies have shown that lower topographic relief, and higher infiltration rates, coupled with increased transmissivity of material from waste rock dumps (i.e., valley fill) can alter the flow paths and residence time of water within mined watersheds.^4^ What is surprising is that we can observe this through changing solute dynamics 80–120 km downriver from the ERM, at the outflow of the larger Elk River Watershed, indicating the profundity of the change that large scale mining is having on solute transport.

The spring freshet is still the period when the largest mass of solutes is delivered to KOC. However, the baseflow is the time when the highest concentration waters flow into the reservoir. Based on the patterns we have observed in the changes in solute dynamics, increasing transient storage and solute generation from the growing volume of waste rock has raised baseflow solute concentrations more than those occurring at other times in the year, which may increase further with additional mining. High base-flow concentrations are likely to persist unless water treatment can increase and outpace growing solute delivery from additional mining and address all major sources. Our analyses suggest that current ERM water treatment has the potential to decrease concentrations in the Elk River during low discharge portions of the year; however, treatment will have much less effect on the annual loads being delivered to KOC—meaning how solute concentrations are distributed in time and space within the reservoir may have significant implications for downstream ecosystems, fisheries, and water-quality targets. This is an important finding that could assist in the management of beneficial uses in KOC because the mass of contaminants being delivered to KOC post ERM water treatment may not have the same proportional reduction that annual average concentrations may have. Likewise, there is limited primary literature on the effects on the aquatic ecosystem in KOC thus far. But this work is a first step in quantifying masses of solutes entering the reservoir over time, which provides context for ecological studies going forward.

Mining operations have changed the solute delivery dynamics within the Elk River Watershed between 1984 and 2022, while the Kootenay River has remained largely unchanged. With the introduction of water treatment and planned treatment expansions, understanding how treatment further changes the hydrologic and geochemical systems in conjunction with planned and proposed mining operations may support management decision making. There are still many areas that could benefit from further research, including: surface water–groundwater interaction in the Elk Valley and its mine-affected tributaries, an understanding of the magnitude and extent of groundwater contamination, the long-range transport potential of Se, and a better understanding of how treatment will affect downstream concentrations and loads. In addition, an assessment of the Se and NO_3_^–^ mass balance in KOC is needed. Including developing an understanding of spatial and temporal distribution of contaminants within KOC, both current and historic, which may improve understanding of the downstream effects on solute concentrations and loads from mining operations and associated water treatment—an important consideration given the potential for effects to aquatic ecosystems and fisheries. Given current and planned mine operations and water treatment, it is unclear whether current and planned surface water treatment will be sufficient to meet downstream water-quality regulations. This transboundary system presents a unique challenge for managers and decision makers on both sides of the border and has implications for water quality throughout the Kootenai Basin and the broader Columbia River system.

## Supporting Information Available

Method details, additional site information, WRTDS model governing equation, and additional tables and figures (PDF)

## Abbreviations

KOC: Koocanusa Reservoir
ERM: Elk River Mines
RWQM: Teck Resources Regional Water Quality model
WRTDS: weighted regression on time discharge and season
MT: Montana
ID: Idaho
USGS: United States Geologic Survey
B.C.: British Columbia
CA: Canada
U.S.: United States
ECCC: Environment and Climate Change Canada

## References

[ref1] Cooley H.; Gleick P. H. Climate-proofing transboundary water agreements. Hydrological Sciences Journal 2011, 56 (4), 711–718. 10.1080/02626667.2011.576651.

[ref2] Wolf A. T. Criteria for equitable allocations: The heart of international water conflict. Natural Resources Forum 1999, 23 (1), 3–30. 10.1111/j.1477-8947.1999.tb00235.x.

[ref3] Puri S.; Aureli A. Atlas of transboundary aquifers: Global maps, regional cooperation and local inventories; ISARM Program: 2009.

[ref4] Nippgen F.; Ross M. R. V.; Bernhardt E. S.; McGlynn B. L. Creating a more perennial problem? Mountaintop removal coal mining enhances and sustains saline baseflows of Appalachian watersheds. Environ. Sci. Technol. 2017, 51 (15), 8324–8334. 10.1021/acs.est.7b02288. 28704046

[ref5] Wellen C.; Shatilla N. J.; Carey S. K. The influence of mining on hydrology and solute transport in the Elk Valley, British Columbia, Canada. Environmental Research Letters 2018, 13 (7), 074012 10.1088/1748-9326/aaca9d.

[ref6] Meredith E.; Wheaton J.; Kuzara S. Hydrogeologic responses to 50 years of surface coal mining and 20 years of coalbed-methane production in southeastern Montana with an emphasis on reclamation at Big Sky Mine. Special Publication 122: Geology of Montana; Montana Bureau of Mines and Geology: 2021; https://www.mbmg.mtech.edu/pubs/GeologyofMontana/#gsc.tab=0 (accessed March 22, 2023).

[ref7] Palmer M. A.; Bernhardt E. S.; Schlesinger W. H.; Eshleman K. N.; Foufoula-Georgiou E.; Hendryx M. S.; Lemly A. D.; Likens G. E.; Loucks O. L.; Power M. E.; et al. Mountaintop mining consequences. Science 2010, 327 (5962), 148–149. 10.1126/science.1180543. 20056876

[ref8] Sergeant C. J.; Sexton E. K.; Moore J. W.; Westwood A. R.; Nagorski S. A.; Ebersole J. L.; Chambers D. M.; O’Neal S. L.; Malison R. L.; Hauer F. R. Risks of mining to salmonid-bearing watersheds. Science Advances 2022, 8 ( (26), ); 10.1126/sciadv.abn0929. PMC10883362 35776798

[ref9] Who We Are. Ktunaxa Nation Home Page. 2023. https://www.ktunaxa.org/who-we-are/ (accessed December 2022).

[ref10] Presser T. S.; Naftz D. L. Understanding and documenting the scientific basis of selenium ecological protection in support of site-specific guidelines development for Lake Koocanusa, Montana, U.S.A., and British Columbia, Canada; U.S. Geological Survey Open-File Report 2020-1098; U.S. Geological Survey: Reston, VA, 2020; 10.3133/ofr20201098.

[ref11] Government of Canada. Provinces/territories, cartographic boundary file-2016 census. 2023. https://open.canada.ca/data/en/dataset/a883eb14-0c0e-45c4-b8c4-b54c4a819edb (accessed June 22, 2023).

[ref12] U.S. Census Bureau. Cartographic boundary files-Shapefile. 2023. https://www.census.gov/geographies/mapping-files/time-series/geo/carto-boundary-file.2015.html#list-tab-1556094155 (accessed June 22, 2023).

[ref13] Mahmood F. N.; Barbour S. L.; Kennedy C.; Hendry M. J. Nitrate release from waste rock dumps in the Elk Valley, British Columbia, Canada. Science of The Total Environment 2017, 605–606, 915–928. 10.1016/j.scitotenv.2017.05.253. 28693108

[ref14] Wellen C. C.; Shatilla N. J.; Carey S. K. Regional scale selenium loading associated with surface coal mining, Elk Valley, British Columbia, Canada. Science of The Total Environment 2015, 532, 791–802. 10.1016/j.scitotenv.2015.06.040. 26136156

[ref15] Hauer F. R.; Sexton E. K. Transboundary Flathead River: Water quality and aquatic life use final report; Flathead Lake Biological Station, The University of Montana: Polson, MT, 2013; https://files.cfc.umt.edu/cesu/NPS/UMT/2008/08Hauer_GLAC_water_quality_fnlrpt.pdf (accessed December 2022).

[ref16] Cooke C. A.; Drevnick P. E. Transboundary atmospheric pollution from mountaintop coal mining. Environmental Science & Technology Letters 2022, 9 (11), 943–948. 10.1021/acs.estlett.2c00677.

[ref17] Essilfie-Dughan J.; Hendry M. J.; Dynes J. J.; Hu Y.; Biswas A.; Lee Barbour S.; Day S. Geochemical and mineralogical characterization of sulfur and iron in coal waste rock, Elk Valley, British Columbia, Canada. Sci. Total Environ. 2017, 586, 753–769. 10.1016/j.scitotenv.2017.02.053. 28202241

[ref18] Ross M. R.; McGlynn B. L.; Bernhardt E. S. Deep Impact: Effects of Mountaintop Mining on Surface Topography, Bedrock Structure, and Downstream Waters. Environ. Sci. Technol. 2016, 50 (4), 2064–2074. 10.1021/acs.est.5b04532. 26800154

[ref19] Lindberg T. T.; Bernhardt E. S.; Bier R.; Helton A. M.; Merola R. B.; Vengosh A.; Di Giulio R. T. Cumulative impacts of mountaintop mining on an Appalachian watershed. Proc. Natl. Acad. Sci. U. S. A. 2011, 108 (52), 20929–20934. 10.1073/pnas.1112381108. 22160676 PMC3248525

[ref20] Brooks A. C.; Ross M. R. V.; Nippgen F.; McGlynn B. L.; Bernhardt E. S. Excess Nitrate Export in Mountaintop Removal Coal Mining Watersheds. Journal of Geophysical Research: Biogeosciences 2019, 124 (12), 3867–3880. 10.1029/2019JG005174.

[ref21] Welch C.; Barbour S. L.; Hendry M. J. The geochemistry and hydrology of coal waste rock dumps: A systematic global review. Science of The Total Environment 2021, 795, 148798 10.1016/j.scitotenv.2021.148798. 34247080

[ref22] Lange D. A.; Storb M. B. Input files and WRTDS model output for the two major tributaries of Lake Koocanusa: U.S. Geological Survey Data Release 2023, 10.5066/P9JP8VGL.

[ref23] British Columbia Ministry of Forests Lands Natural Resource Operations and Rural Development. BC Water Tool - Watershed. 2023. https://kwt.bcwatertool.ca/watershed (accessed February 9, 2023).

[ref24] U.S. Geological Survey. USGS water data for the Nation: U.S. Geological Survey National Water Information System database; 2023 (accessed May 25, 2023); 10.5066/F7P55KJN.

[ref25] Jamieson H. E. Geochemistry and mineralogy of solid mine waste: Essential knowledge for predicting environmental impact. Elements 2011, 7 (6), 381–386. 10.2113/gselements.7.6.381.

[ref26] Huggins F. E.; Seidu L. B. A.; Shah N.; Huffman G. P.; Honaker R. Q.; Kyger J. R.; Higgins B. L.; Robertson J. D.; Pal S.; Seehra M. S. Elemental modes of occurrence in an Illinois #6 coal and fractions prepared by physical separation techniques at a coal preparation plant. International Journal of Coal Geology 2009, 78 (1), 65–76. 10.1016/j.coal.2008.10.002.

[ref27] Lussier C.; Veiga V.; Baldwin S. The geochemistry of selenium associated with coal waste in the Elk River Valley, Canada. Environmental Geology 2003, 44 (8), 905–913. 10.1007/s00254-003-0833-y.

[ref28] Ross M. R. V.; Nippgen F.; Hassett B. A.; McGlynn B. L.; Bernhardt E. S. Pyrite Oxidation Drives Exceptionally High Weathering Rates and Geologic CO2 Release in Mountaintop-Mined Landscapes. Global Biogeochem Cy 2018, 32 (8), 1182–1194. 10.1029/2017GB005798.

[ref29] Teck Resources Limited. Elk Valley water quality plan: 2022 implementation plan adjustment; 2022. https://www.teck.com/media/EVWQP_2022_ImplementationPlanAdjustment_Main_Report.pdf (accessed January 9, 2023).

[ref30] Chowanski K.; Kunza L.; Hoffman G.; Genzoli L.; Stickney E. River management alters ecosystem metabolism in a large oligotrophic river. Freshwater Science 2020, 39 (3), 534–548. 10.1086/710082.

[ref31] Cianciolo T. R.; McLaughlin D. L.; Zipper C. E.; Timpano A. J.; Soucek D. J.; Whitmore K. M.; Schoenholtz S. H. Selenium bioaccumulation across trophic levels and along a longitudinal gradient in headwater streams. Environ. Toxicol. Chem. 2020, 39 (3), 692–704. 10.1002/etc.4660. 31900941

[ref32] Elser J. J.; Dobberfuhl D. R.; MacKay N. A.; Schampel J. H. Organism size, life history, and N:P stoichiometry: Toward a unified view of cellular and ecosystem processes. BioScience 1996, 46 (9), 674–684. 10.2307/1312897.

[ref33] Elser J. J.; Andersen T.; Baron J. S.; Bergström A. K.; Jansson M.; Kyle M.; Nydick K. R.; Steger L.; Hessen D. O. Shifts in lake N:P stoichiometry and nutrient limitation driven by atmospheric nitrogen deposition. Science 2009, 326 (5954), 835–837. 10.1126/science.1176199. 19892979

[ref34] Stickney E.; Kunza L.; Hoffman G.; Chowanski K. Small land cover changes in the transboundary Kootenai River basin greatly alter water quality. River Research and Applications 2021, 37 (4), 605–614. 10.1002/rra.3783.

[ref35] Tan L. C.; Nancharaiah Y. V.; van Hullebusch E. D.; Lens P. N. L. Selenium: environmental significance, pollution, and biological treatment technologies. Biotechnology Advances 2016, 34 (5), 886–907. 10.1016/j.biotechadv.2016.05.005. 27235190

[ref36] Etteieb S.; Magdouli S.; Zolfaghari M.; Brar S. Monitoring and analysis of selenium as an emerging contaminant in mining industry: A critical review. Science of The Total Environment 2020, 698, 134339 10.1016/j.scitotenv.2019.134339. 31783461

[ref37] Hamilton S. J. Review of selenium toxicity in the aquatic food chain. Science of The Total Environment 2004, 326 (1), 1–31. 10.1016/j.scitotenv.2004.01.019. 15142762

[ref38] English S. G.; Hess H.; Bishop C. A.; Porter E.; Cheng K. M.; Elliott J. E. Bioaccumulation and effects of selenium from surface coal mining in an aquatic songbird. Environmental Research 2022, 208, 112702 10.1016/j.envres.2022.112702. 35026185

[ref39] Lemly A. D. Selenium impacts on fish: an insidious time bomb. Human and Ecological Risk Assessment 1999, 5 (6), 1139–1151. 10.1080/10807039.1999.10518883.

[ref40] Montana Department of Environmental Quality. Final 2018 Water Quality Integrated Report; 2018. https://deq.mt.gov/files/Water/WQPB/CWAIC/Reports/IRs/2018/2018_IR_Final.pdf (accessed April 4, 2022).

[ref41] Idaho Department of Environmental Quality. Idaho’s 2018/2020 integrated report; State of Idaho Department of Environmental Quality: 2020. https://www2.deq.idaho.gov/admin/LEIA/api/document/download/14888 (accessed March 22, 2023).

[ref42] Idaho Department of Environmental Quality. Idaho’s 2022 integrated report; State of Idaho Department of Environmental Quality, 2022. https://www2.deq.idaho.gov/admin/LEIA/api/document/download/16619 (accessed June 21, 2023).

[ref43] Administrative Rules of Montana. Selenium standards for Lake Koocanusa and the Kootenai River; Montana Department of Environmental Quality: 2020. http://www.mtrules.org/gateway/RuleNo.asp?RN=17%2E30%2E632 (accessed February 9, 2023).

[ref44] Idaho Department of Environmental Quality. IDAPA 58.01.02 - Water quality standards; Idaho Department of Environmental Quality, Surface Water Division: 2023. https://adminrules.idaho.gov/rules/current/58/580102.pdf (accessed June 21, 2023).

[ref45] Montana Department of Environmental Quality. Derivation of a site-specific water column selenium standard for Lake Koocanusa, Montana; Montana Dept. of Enviornmental Quality: Helena, MT, 2020. https://deq.mt.gov/files/Water/WQPB/Standards/Koocanusa/TSD_Lake%20Koocanusa_Sep2020_Final.pdf (accessed October 3, 2023).

[ref46] Waste discharge authorization: Authorization management system, authorization #107517; https://www2.gov.bc.ca/gov/content/environment/waste-management/waste-discharge-authorization/find-authorization#searchdocuments (accessed June 1, 2023).

[ref47] British Columbia Ministry of Environment. Ambient water quality guidelines for selenium technical report update; Water Protection and Sustainability Branch: 2014. https://www2.gov.bc.ca/assets/gov/environment/air-land-water/water/waterquality/water-quality-guidelines/approved-wqgs/bc_moe_se_wqg.pdf (accessed October 3, 2023).

[ref48] Hirsch R. M.; Moyer D. L.; Archfield S. A. Weighted Regressions on Time, Discharge, and Season (WRTDS), with an application to Chesapeake Bay River inputs. Journal of the American Water Resources Association 2010, 46 (5), 857–880. 10.1111/j.1752-1688.2010.00482.x. 22457569 PMC3307614

[ref49] Cruickshank A. How pollution from Canadian coal mines threatens the fish at the heart of communities from B.C. to Idaho; The Narwhal: Victoria, B.C., Canada, 2022. https://thenarwhal.ca/teck-resources-coal-transboundary/ (accessed October 3, 2023).

[ref50] Lemly A. D. Review of Environment Canada’s Teck Coal environmental assessment and evaluation of selenium toxicology tests on Westslope Cutthroat in the Elk and Fording Rivers in Souteast British Columbia; Environment Canada Enforcement Division: 2014.

[ref51] Weber B. U.S. wants Canada to join investigation of cross-border pollution from B.C. coal mines; The Canadian Press: 2022. https://www.cbc.ca/news/canada/british-columbia/us-canada-cross-border-pollution-investigation-bc-mines-1.6489348 (accessed September 27, 2023).

[ref52] Robbins J. Tracing mining’s threat to U.S. waters. New York Times; July 11, 2023. https://www.nytimes.com/2023/07/11/science/us-canada-mining-pollution.html (accessed July 12, 2023).

[ref53] U.S. Army Corps of Engineers. Environmental statement: Libby Dam and Lake Koocanusa, Kootenai River, Montana Final Draft, U.S. Army Corps of Engineers, Seattle, Washington; 1972. https://usace.contentdm.oclc.org/digital/api/collection/p16021coll7/id/10891/download (accessed February 9, 2023).

[ref54] Chisholm I.; Hamlin P. Libby reservoir angler census: May 13–October 31, 1987 [i.e. 1985]: Interim Report; Montana Department of Fish, Wildlife and Parks: Kalispell, MT, 1987. https://www.osti.gov/biblio/5494223 (accessed December 2022). 10.2172/5494223.

[ref55] British Columbia Ministry of Forests Lands Natural Resource Operations and Rural Development. B.C. Water Tool - Streamflow & Water Levels; 2023. https://kwt.bcwatertool.ca/streamflow (accessed June 6, 2023).

[ref56] British Columbia Ministry of Forests Lands Natural Resource Operations and Rural Development. Elk Valley Water Quality Hub - Website; 2023. https://elkvalleywaterquality.gov.bc.ca/ (accessed May 16, 2023).

[ref57] Clarke G.; Northcote B.; Katay F.; Tombe S. P. Exploration and mining in British Columbia, 2020: A summary. In Provincial Overview of Exploration and Mining in British Columbia, 2020; British Columbia Geological Survey Information Circular 2021–-01, British Columbia Ministry of Energy, Mines and Low Carbon Innovation: 2021; pp 1–45.

[ref58] Teck Coal Limited. 2020 regional water quality model update report; 2021. https://www.teck.com/media/Teck-EVWQP-2020-RWQM-Update-Report.pdf (accessed December 2022).

[ref59] Golder Associates, Ltd. Elk Valley Water Quality Plan: Annex D.1 Water quality modelling methods; 2014. https://www2.gov.bc.ca/assets/gov/environment/waste-management/industrial-waste/industrial-waste/mining-smelt-energy/area-based-man-plan/annexes/d1_water_quality_modelling_methods.pdf (accessed June 1, 2023).

[ref60] Villeneuve S. A.; Barbour S. L.; Hendry M. J.; Carey S. K. Estimates of water and solute release from a coal waste rock dump in the Elk Valley, British Columbia, Canada. Sci. Total Environ. 2017, 601–602, 543–555. 10.1016/j.scitotenv.2017.05.040. 28575832

[ref61] Order of the Minister of Environment: section 89 environmental management act, Ministerial order no. M113; Province of British Columbia: 2013.

[ref62] Government of Canada. Columbia River basin long-term water quality monitoring data; 2023. https://open.canada.ca/data/en/dataset/c2adcb27-6d7e-4e97-b546-b8ee3d586aa4/resource/7bb8d1ff-f446-494f-8f3d-ad252162eef5 (accessed February 9, 2023).

[ref63] Hirsch R. M. A comparison of four streamflow record extension techniques. Water Resour. Res. 1982, 18 (4), 1081–1088. 10.1029/WR018i004p01081.

[ref64] Moog D. B.; Whiting P. J.; Thomas R. B. Streamflow record extension using power transformations and application to sediment transport. Water Resour. Res. 1999, 35 (1), 243–254. 10.1029/1998WR900014.

[ref65] Hirsch R. M.; De Cicco L. A. User guide to Exploration and Graphics for RivEr Trends (EGRET) and dataRetrieval: R packages for hydrologic data: U.S. Geological Survey Techniques and Methods 4-A10; USGS: Reston, VA, 2015 (accessed February 9, 2023). 10.3133/tm4A10.

[ref66] RStudio Team. RStudio: integrated development environment for R; RStudio, PBC: Boston, MA, 2021. http://www.rstudio.com/ (accessed January 12, 2023).

[ref67] Choquette A. F.; Hirsch R. M.; Murphy J. C.; Johnson L. T.; Confesor R. B. Tracking changes in nutrient delivery to western Lake Erie: Approaches to compensate for variability and trends in streamflow. Journal of Great Lakes Research 2019, 45 (1), 21–39. 10.1016/j.jglr.2018.11.012.

[ref68] Helsel D. R.; Hirsch R. M.; Ryberg K. R.; Archfield S. A.; Gilroy E. J. Statistical methods in water resources; USGS: Reston, VA, 2020 (accessed February 13, 2023). 10.3133/tm4A3.

[ref69] Rowland F. E.; Stow C. A.; Johnson L. T.; Hirsch R. M. Lake Erie tributary nutrient trend evaluation: Normalizing concentrations and loads to reduce flow variability. Ecological Indicators 2021, 125, 107601 10.1016/j.ecolind.2021.107601.

[ref70] Hirsch R. M.; Archfield S. A.; De Cicco L. A. A bootstrap method for estimating uncertainty of water quality trends. Environmental Modelling & Software 2015, 73, 148–166. 10.1016/j.envsoft.2015.07.017.

[ref71] Lee C. J.; Hirsch R. M.; Crawford C. G. An evaluation of methods for computing annual water-quality loads: U.S. Geological Survey Scientific Investigations Report 2019-5084; USGS: Reston, VA, 2019 (accessed February 9, 2023). 10.3133/sir20195084.

[ref72] Zhang Q.; Hirsch R. M. River water-quality concentration and flux estimation can be improved by accounting for serial correlation through an autoregressive model. Water Resour. Res. 2019, 55 (11), 9705–9723. 10.1029/2019WR025338.

[ref73] Zinsser L. M. Trends in concentration, loads, and sources of trace metals and nutrients in the Spokane River Watershed, northern Idaho, water years 1990–2018; USGS: Reston, VA, 2020 (accessed March 22, 2023). 10.3133/sir20205096.

[ref74] Luoma K.; Niemi J. V.; Aurela M.; Fung P. L.; Helin A.; Hussein T.; Kangas L.; Kousa A.; Rönkkö T.; Timonen H.; et al. Spatiotemporal variation and trends in equivalent black carbon in the Helsinki metropolitan area in Finland. Atmospheric Chemistry and Physics 2021, 21 (2), 1173–1189. 10.5194/acp-21-1173-2021.

[ref75] Clow D. W.; Drever J. I. Weathering rates as a function of flow through an alpine soil. Chem. Geol. 1996, 132 (1), 131–141. 10.1016/S0009-2541(96)00048-4.

[ref76] Knapp J. L. A.; von Freyberg J.; Studer B.; Kiewiet L.; Kirchner J. W. Concentration-discharge relationships vary among hydrological events, reflecting differences in event characteristics. Hydrology and Earth System Sciences 2020, 24 (5), 2561–2576. 10.5194/hess-24-2561-2020.

[ref77] Clow D. W.; Mast M. A. Mechanisms for chemostatic behavior in catchments: Implications for CO_2_ consumption by mineral weathering. Chem. Geol. 2010, 269 (1), 40–51. 10.1016/j.chemgeo.2009.09.014.

[ref78] Godsey S. E.; Kirchner J. W. Dynamic, discontinuous stream networks: Hydrologically driven variations in active drainage density, flowing channels and stream order. Hydrological Processes 2014, 28 (23), 5791–5803. 10.1002/hyp.10310.

[ref79] Godsey S.; Kirchner J.; Clow D. Concentration discharge relationships reflect chemostatic characteristics of U.S. catchments. Hydrological Processes 2009, 23, 1844–1864. 10.1002/hyp.7315.

[ref80] Exploration and Graphics for RivEr Trends (EGRET); USGS: 2023. https://pubs.usgs.gov/tm/04/a10/ (accessed Febuary 9, 2023).

[ref81] Murphy S. F.; McCleskey R. B.; Writer J. H. Effects of flow regimes on stream turbidity and suspended solids after wildfire, Colorado Front Range, USA. In Wildfire and Water Quality: Processes, Impacts and Challenges; IAHS Publ. 354; IAHS Press: 2012; p 325.

[ref82] Murphy J. C.; Hornberger G. M.; Liddle R. G. Concentration-discharge relationships in the coal mined region of the New River basin and Indian Fork sub-basin, Tennessee, USA. Hydrological Processes 2014, 28 (3), 718–728. 10.1002/hyp.9603.

[ref83] Katay F. Exploration and mining in the Kootenay-Boundary Region, British Columbia. In: Exploration and Mining in British Columbia, 2014; British Columbia Ministry of Energy and Mines, British Columbia Geological Survey: 2015. https://cmscontent.nrs.gov.bc.ca/geoscience/publicationcatalogue/InformationCircular/BCGS_IC2016-01-03.pdf (accessed February 9, 2023).

[ref84] British Columbia. Canada-B.C. Water Quality Monitoring Program; https://www.nrs.gov.bc.ca/ecms/CanadaBCWQReports/TrendReports/BC08NK0003_SiteReport.html (accessed September 27, 2023).

[ref85] British Columbia Ministry of Environment. Water quality guidelines for nitrogen (nitrate, nitrite, and ammonia); 2009. https://www2.gov.bc.ca/assets/gov/environment/air-land-water/water/waterquality/water-quality-guidelines/approved-wqgs/nitrogen-addendum.pdf#:~:text=For%20nitrate%20%28as%20N%29%2C%20the%2030-d%20average%20concentration,to%20protect%20marine%20aquatic%20life%203.7%2A%20mg%20L-1 (accessed June 28, 2023).

[ref86] British Columbia Ministry of Environment. Ambient water quality guidelines for sulphate; 2013. https://www2.gov.bc.ca/assets/gov/environment/air-land-water/water/waterquality/water-quality-guidelines/approved-wqgs/sulphate/bc_moe_wqg_sulphate.pdf#:~:text=Sulphate%20is%20a%20potentially%20harmful%20contaminant%20in%20freshwater,toxicity%20data%20to%20develop%20a%2030-day%20average%20guideline (accessed June 28, 2023).

[ref87] Environment and Climate Change Canada. Canadian environmental protection act, 1999 federal environmental quality guidelines: Selenium; 2022. https://www.canada.ca/content/dam/eccc/documents/pdf/pded/feqg-selenium/Federal-environmental-quality-guidelines-selenium.pdf (accessed June 1, 2023).

[ref88] Teck Resources Limited. Water quality in the Elk Valley; 2023. https://www.teck.com/sustainability/sustainability-topics/water/water-quality-in-the-elk-valley/ (accessed February 9, 2023).

[ref89] Burri N. M.; Weatherl R.; Moeck C.; Schirmer M. A review of threats to groundwater quality in the anthropocene. Science of The Total Environment 2019, 684, 136–154. 10.1016/j.scitotenv.2019.05.236. 31153063

[ref90] Teck Coal Limited. 2020 annual report; 2020. https://www.teck.com/media/2020-Annual-Report.pdf (accessed February 9, 2023).

[ref91] Ganapathi H.; Phukan M. Environmental hazards of limestone mining and adaptive practices for environment management plan. In Environmental Processes and Management: Tools and Practices; Singh R. M., Shukla P., Singh P., Eds.; Springer International Publishing: 2020; pp 121–134.

[ref92] Conde J. E.; Sanz Alaejos M. Selenium concentrations in natural and environmental waters. Chem. Rev. 1997, 97 (6), 1979–2004. 10.1021/cr960100g. 11848896

[ref93] Pommen Water Quality Consulting. Water quality assessment of St. Mary River at Wycliffe (1973–2003); 2004. https://www2.gov.bc.ca/assets/gov/environment/air-land-water/water/waterquality/monitoring-water-quality/kootenay-wq-docs/wq_ko_stmary_river_wycliffe.pdf (accessed June 1, 2023).

[ref94] Huang M.; Barbour S. L.; Hendry M. J. Simulating nitrate release from an unsaturated coal waste rock dump. Science of The Total Environment 2021, 779, 146429 10.1016/j.scitotenv.2021.146429. 33743462

[ref95] Hendry M. J.; Biswas A.; Essilfie-Dughan J.; Chen N.; Day S. J.; Barbour S. L. Reservoirs of Selenium in Coal Waste Rock: Elk Valley, British Columbia, Canada. Environ. Sci. Technol. 2015, 49 (13), 8228–8236. 10.1021/acs.est.5b01246. 26038975

[ref96] Day S.; Kennedy C.; Pumphrey J. Interpretation of selenium release from coal mine waste rock dumps, Southeastern British Columbia, Canada, 9th International Conference on Acid Rock Drainage, Ottawa, Canada, 2012.

[ref97] Read E. K.; Carr L.; De Cicco L.; Dugan H. A.; Hanson P. C.; Hart J. A.; Kreft J.; Read J. S.; Winslow L. A. Water quality data for national-scale aquatic research: The Water Quality Portal. Water Resour. Res. 2017, 53 (2), 1735–1745. 10.1002/2016WR019993.

