# Supplementary material for: Growth of Coal Mining Operations in the Elk River Valley (Canada) Linked to Increasing Solute Transport of Se, NO3–, and SO42– into the Transboundary Koocanusa Reservoir (USA–Canada)
Source: Environ Sci Technol. 2023 Nov 3;57(45):17465–80. doi: 10.1021/acs.est.3c05090 (PMC10653222; doi:10.1021/acs.est.3c05090)
Supplement: Supplementary file 1 — es3c05090_si_001.pdf [file es3c05090_si_001.pdf]

Supporting Information for:

Growth of coal mining operations in the Elk River Valley (B.C. Canada) linked to increasing solute transport of Se, NO_3^-, and SO_4^{2-} into the transboundary Koocanusa Reservoir (USA-Canada)

Meryl B. Storb[1,3*], Ashley M. Bussell[1], Sara L. Caldwell Eldridge[1], Robert M. Hirsch[2], Travis S. Schmidt[1]

1. U.S. Geological Survey WY- MT Water Science Center, 3162 Bozeman Avenue, Helena MT 59601

2. U.S. Geological Survey Water Mission Area, 12201 Sunrise Valley Dr, Reston, VA 20192

3. Department of Land Resources and Environmental Sciences, Montana State University, Bozeman, Montana, USA

Journal: Environmental Science and Technology
Prepared June 2023
29 pp. in length, including text, 5 tables and 13 figures.

Methods

SI Geospatial Analysis for Mine Area

Total area of disturbance (waste rock deposits) was estimated using remote sensing and geospatial analysis techniques (SI Figure 1). Specifically, Sentinel 2 data [1] for August, 2022 were mosaicked and composited for the Elk River Watershed using Google Earth Engine. [2] A function to mask clouds from the image using bands 10 and 11 was applied and pixel values for all images included in the composite were reduced to the median value for each band. Random forest classification was then done on the composite image. [3] The classification model was trained with ten trees using the training classes and associated sample sizes shown in table SI 1a. Fifty percent of the training data were held out for validation for each tree. Total mean error from validation on holdout sites was 12.7%. The classified image was then imported to ArcPro[4] for postprocessing. The image was converted to a multipart polygon using the conversion tools. Next, the Region Group tool was used to eliminate polygons less than 5,000 square meters, as these small polygons likely represented misclassified pixels rather than actual landcover features. Additionally, because the objective of this exercise was to extract the perimeter of the waste rock area, small polygons located within the waste rock polygons associated with a class other than waste rock were eliminated on the assumption that while the surface reflectance in the imagery might indicate the presence of another landcover (e.g., water or vegetation) it is likely still underlain by waste rock. The final areas of waste rock are shown in table SI 1b.

Additional site information

The Kootenay River near Fenwick Station (BC08NG0009) is 13 km downstream of the Fort Steele discharge station (08NG065) and downstream of the confluence with the Saint Mary's River, but upstream of the confluence with the Bull River (Figure 1). [5] Sample collection at this

site was discontinued in September 2019. The mean daily discharge for the Kootenay at Fort Steele (WSC site 08NG065) between 1963 and 2022 was 148 m^3sec^{-1}. [5] Because the discharge and water sampling locations are not concurrent, flow was corrected by the difference in contributing area between the two sites, meaning flow at the Fort Steele location was increased by 2.82% to estimate flows at the Fenwick location. Adjusted flows for the Fenwick site are available in Lange and Storb [6]

There is a historical Water Survey of Canada (WSC) site the Elk River at Phillips Bridge (08NK005), 6.7 km upstream of the Highway 93 sampling location[5]. Flow at Phillips Bridge was measured from 1924-1996. The closest flow measurement location that is currently in operation is the WSC Elk River at Fernie site (08NK002). Thus, Move2 streamflow record extension techniques [7, 8] were implemented in R[55] using the smwrStats package[9] to extend the daily flow record at Phillips Bridge, based on the flow relationship between the two sites post 1970 (Correlation coefficient 0.9805). Once the record extension was complete, an area correction factor was applied to account for the difference in contributing area between the Phillips Bridge and the Highway 93 sampling location, flows from Phillips Bridge were increased by 0.49%. Adjusted flows for the Highway 93 site are available in Lange and Storb [6]

Geology

The Elk and Kootenay Watersheds are both contained within the Canadian Cordillera and bisected by faults that run north to south. In both watersheds faults have generated the valley bottoms where their respective rivers are present, however the bedrock geologies are different. [10] The Upper Kootenay Watershed is generally comprised of carbonate and silicate geology, the eastern side is primarily limestones and dolomites, and the western side is dominated by carbonate

and siliciclastic formations. The Kootenay River overlays quaternary alluvium, and the riverbed mirrors the Rocky Mountain Trench fault [10]. The Elk River Watershed generally runs north to south, with the Elk River overlaying a quaternary alluvial aquifer and mirrors the Bourgeau thrust fault from its northern extent to the town of Fernie. The Elk Valley contains two of the three, structurally separate, major coalfields in British Columbia (B.C.) [11]. Mining in the Elk River Watershed is focused on bituminous coal from the Mist Mountain Formation which is part of the Jurassic-Cretaceous Kootenay Group (deposited 150-130 M years ago; 450-550 m thick) and is underlain by limestone bedrock, which has high karst potential in some areas [12, 13].

Coal has been mined in the Elk River Watershed since 1897, with large-scale mining in the valley beginning in the 1970's with the transition to open pit drill and blast methods [14]. The Kootenay River Watershed is also home to mining operations, including a smelter and several gypsum and silica mines. Several metal explorations are also occurring [10] and the Sullivan Metal Mine (operated by Teck Resources for Pb, Zn and silver (Ag) production) near the town of Kimberly operated for almost a century, but closed in 2001. [15]

Analysis methods

WRTDS Model Governing Equation

The Weighted Regression on Time Discharge and Seasons (WRTDS) model is based on using statistical smoothing by partitioning the variation present in constituent concentration values into three components and an error term. The four components are related to season within the year, the watershed hydrologic condition or discharge component, long-term trend, and the random unexplained portion of the variation [16]. The basic form of the underlying WRTDS model is below, where c is concentration; Q is discharge; t is time in years; and ε is unexplained variation:

$$\ln(c) = \beta 0 + \beta 1\ t + \beta 2 \ln(Q) + \beta 3 \sin(2\pi t) + \beta 4 \cos(2\pi t) + \varepsilon \quad (1)$$

The equation is a weighted regression and is fit in the form of a weighted Tobit model (i.e., survival regression). The model accommodates the incorporation of non-detect data because each concentration value can be expressed as a single number for a data point with a detection or as an interval between 0 and the reporting limit for non-detect values [17, 18].

Likelihoods were determined from 250 bootstrap intervals assuming stationary flow normalization.

Kalman

Performance of WRTDS_Kalman[19] depends on the AR1 coefficient (ρ), and that relationship varies with constituents and sampling scenarios. The default for ρ within the WRTDS_Kalman function is 0.9 [19, 20]. This was utilized for selenium and sulfate after exploration of larger and smaller ρ values (0.85 and 0.95) did not generate estimates that were substantially different (<5%), ρ = 0.95 was used for nitrate for WRTDS_Kalman[19] estimates for both rivers following results presented in Zhang and Hirsch[19].

Exceedance probability

One way of describing the trends in Se concentrations over the period of record is to use estimates of the expected number days in each year when Se concentrations exceeded the water quality criteria. These calculations are made by using the WRTDS model of Se for the Elk River. For each day of the 38-year long record[6] the WRTDS model provides an estimate of conditional mean and standard deviation of the natural log of concentration for that day (conditioned on year, time of year, and discharge). Using the observed residuals from the fitted WRTDS model as a representation of the probability distribution of the standardized residual for each day, we estimate, for each day in the period of record, the probability of exceedance of the criteria. The

expected number of days that concentration exceeded the criteria is simply the sum of the probabilities for all days in the year.

The approach to calculating the expected number of days on which the Se concentrations exceed a specified criterion is based on the estimated WRTDS model and the discharge record.

Let x_i = the concentration on day i,

$y_i = \ln(x)_i$

$\bar{y}_i$ = the estimated conditional mean of y_i from the WRTDS model

s_i = the standard deviation of the distribution of y_i from the WRTDS model

where i is the index of all 14,004 days in sequence, starting with 1984-07-10 and ending with 2022-11-11.

We can express the value if y_i on any given day as:

$$y_i = \bar{y}_i + s_i \cdot e_i$$

The e_i values are standardized residuals, computed from the WRTDS model and the data set of 774 observed concentrations. They are computed as:

$$e_j = \frac{y_j - \bar{y}_j}{s_j} \quad \text{for } j = 1, 2, \dots, 774$$

The subscript j here refers to the sequence number of sample values, (1 to 774).

Rather than using some theoretical distribution (such as gaussian) for these standardized residuals we use the population of observed standardized residuals from the data set. [6] In the case considered here, the Elk River Se data, we have 774 observations and hence 774 observed

standardized residuals (these are a set of jack-knife cross-validation estimates computed in the modelEstimation() function in EGRET).[18] It is assumed here that each of these 774 residuals is equally likely to have occurred on any given day in the 14,004-day record.

For purposes of estimation of the probability of exceedance of the water quality criterion, denoted here as x^*. The natural log of the criterion is denoted as $y^* = \ln(x^*)$.

For each day in the period of record ($i = 1, 2, \ldots 14004$) we compute 774 equally likely outcomes one for each sampled day ($j = 1, 2, \ldots\ 774$)). Those 10,839,096 ($= 14004 \cdot 774$) outcomes are denoted $V_{i,j}$ and they are computed as:

$$V_{i,j} = \begin{Bmatrix} 1\ if\ \bar{y}_i + s_i \cdot e_j\ \geq y^* \\ 0\ if\ \bar{y}_i + s_i \cdot e_j\ < y^* \end{Bmatrix}$$

Then the estimated probability that concentration on day i exceeds the criterion x^* is defined as

$$p_i\ = \frac{\sum_{j=1}^{774} V_{i,j}}{774} \qquad \text{for } i = 1, 2, \ldots, 14004$$

We can then compute the expected number of exceedances for each water year in the record as $z_k = \sum_{i \in U_k} p_i$

Where, $i \in U_k$ denotes the set of all days, i, in year k.

The z_k represent the expected number of days of exceedances in year k and can take on any value from 0 to 365 (or 366 in a leap year).

Any use of trade, firm, or product names is for descriptive purposes only and does not imply endorsement by the U.S. Government.

SI Figure 1

Total area of mine disturbance (waste rock deposits) based on GIS analysis of aerial imagery.

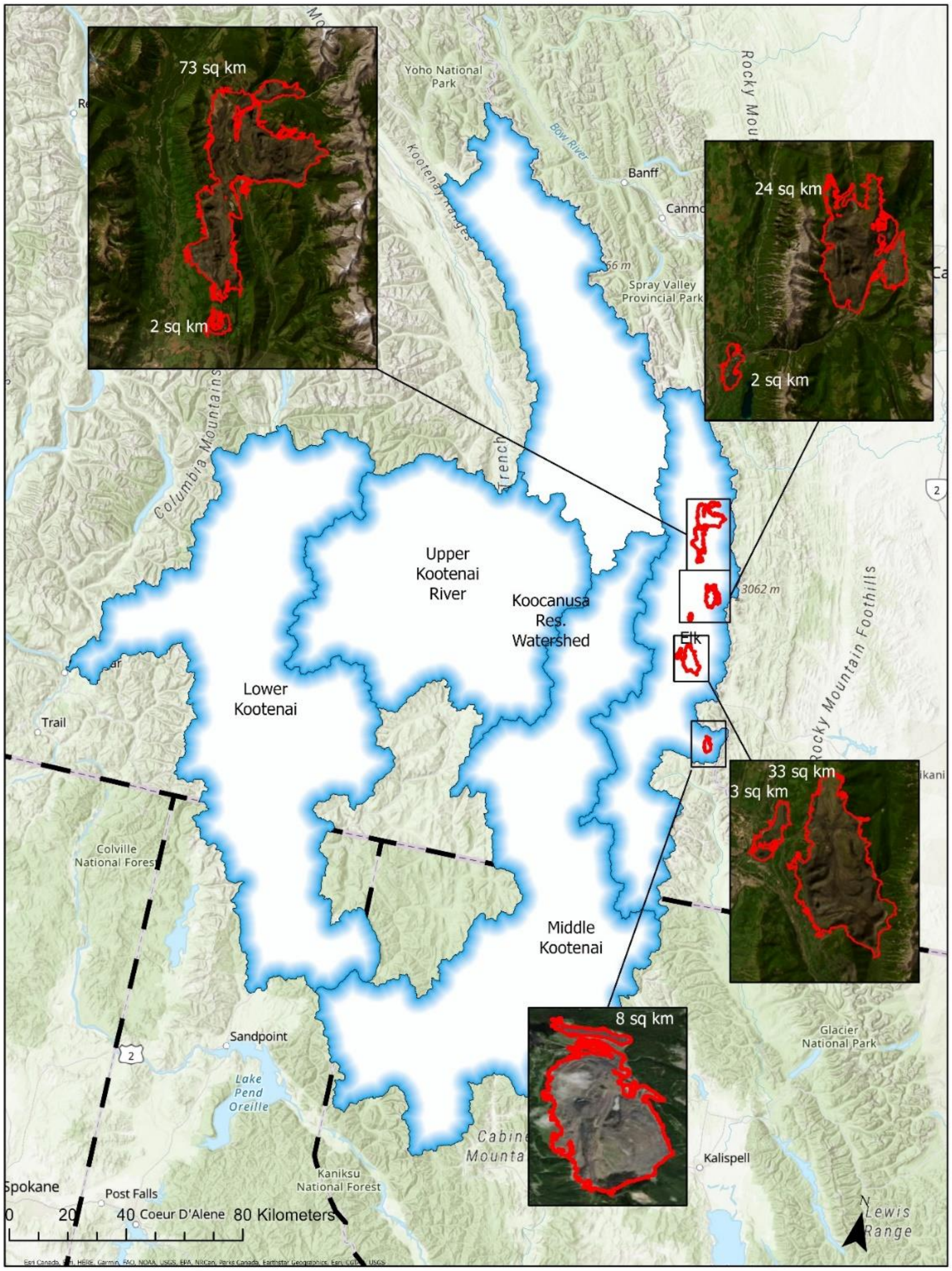

SI Table 1a

Training classes and associated sample sizes from the GIS analysis of mine waste rock area

Sample size (n)	Class
228	Waste rock
89	Forest
68	Water
81	Open rock
60	Deforested

SI Table 1b

Final areas of waste rock from GIS analysis.

Mine	Area of disturbance, in square kilometers
Fording River Operations	75 km^2
Line Creek Operations	26 km^2
Elkview Operations	36 km^2
Coal Mountain Operations (in closure period)	8 km^2

SI Table 2

Elk River Watershed cumulative waste rock volumes by mine, reported by Teck Resources in their 2022 Implementation Plan Adjustment Document (Table 2.1).[21] Existing volumes are values at the end of 2020 in millions of bank (in situ) cubic meters.

Operation	Existing Volume (BCM x 10^6)	Permitted Volume (BCM x 106) at the end of mining
Fording River	3,036	4,787
Greenhills	808	1,186
Line Creek	797	1,445
Elkview	1,787	3,304
Coal Mountain[1]	311	311
**Total**	**6,739**	**11,033**

1. No longer operating and currently in a closure period.

SI Table 3

Se, NO_3^-, SO_4^{2-} annual estimates of load (optimized for accuracy) for the Elk River (Elk) and Kootenay River (Koot). The proportion that the Elk River contributed by year, from the combined estimates of the two tributaries is shown in percent for each constituent and discharge.[16]

Year	Koot Flow (m^3/sec)	Elk Flow (m^3/sec)	Elk proportion of total combined (%)	Koot Se (t/Yr)	Elk Se (t/Yr)	Elk proportion of total combined (%)	Koot NO_3^- (t/Yr)	Elk NO_3^- (t/Yr)	Elk proportion of total combined (%)	Koot SO_4^{2-} (t/Yr)	Elk SO_4^{2-} (t/Yr)	Elk proportion of total combined (%)
1979	128	58.8	31.50%				442.77	218	33%			
1980	173	67.3	28.00%				557.15	243	30%			
1981	214	90.3	29.70%				713.84	400	36%			
1982	189	72.5	27.70%				672.67	350	34%			
1983	173	67.8	28.20%				581.46	250	30%			
1984	154	60.5	28.20%				594.72	280	32%			
1985	142	61.6	30.30%		1.66		552.34	317	36%	103186	32077	24%
1986	188	75.8	28.70%		2.22		630.72	345	35%	123833	41149	25%
1987	154	61.1	28.40%		2.04		521.04	337	39%	115952	38303	25%
1988	139	52.1	27.30%		1.63		530.82	261	33%	104826	32286	24%
1989	166	63.1	27.50%		1.86		668.4	301	31%	114991	37871	25%
1990	204	89.8	30.60%		3.57		828.57	624	43%	126769	46310	27%
1991	238	100.4	29.70%		4.09		953.65	828	46%	141380	54032	28%
1992	143	53.6	27.30%		2.2		533.41	419	44%	104216	38947	27%
1993	159	67.9	29.90%		3.12		535.13	550	51%	112542	43943	28%
1994	158	61.6	28.10%		3.23		607.38	603	50%	119986	44531	27%
1995	171	76.5	30.90%		3.69		632.1	670	51%	122786	48108	28%
1996	228	101	30.70%		5.44		813.86	893	52%	153292	61970	29%
1997	181	80	30.70%		4.48		589.91	612	51%	132329	51921	28%
1998	175	75.9	30.30%		4.62		634.14	805	56%	136417	54786	29%
1999	220	78.7	26.30%		4.21			794			55958	
2000	185	70.8	27.70%		4.31			627			55155	
2001	108	39.6	26.80%		2.49			356			38955	
2002	169	84.3	33.30%		5.27			679			59197	
2003	133	64	32.50%	0.49	5.22	91.50%		672			58338	
2004	148	59.7	28.70%	0.36	4.22	92.10%		497			50190	
2005	168	77.6	31.60%	0.46	6.1	93.00%		811			62462	
2006	187	87.8	32.00%	0.49	7.83	94.20%		1214			73056	
2007	203	82.2	28.80%	0.56	7.78	93.30%		1115			69321	
2008	160	67.2	29.60%	0.4	6.88	94.50%		1153			63140	
2009	139	49.7	26.30%	0.4	5.54	93.30%	414.02	899	68%		50131	
2010	138	55.6	28.70%	0.38	6.05	94.10%	427.55	1104	72%		54625	
2011	204	80.8	28.40%	0.5	8.48	94.40%	596.62	1633	73%		66141	
2012	238	100.1	29.60%	0.58	11.53	95.20%	712.9	2139	75%		79029	
2013	212	98.9	31.80%	0.49	12.6	96.20%	657.21	2645	80%	135270	88804	40%
2014	192	86.6	31.10%	0.44	12.4	96.60%	589.34	2815	83%	128185	85490	40%
2015	152	63.6	29.50%	0.35	9.99	96.60%	494.69	2038	80%	117210	74140	39%
2016	162	60.9	27.30%	0.42	9.12	95.60%	539.4	2031	79%	127970	76617	37%
2017	210	80.5	27.70%	0.51	9.96	95.20%	848.21	2115	71%	146569	79502	35%
2018	179	70.3	28.20%	0.45	10.21	95.80%	595.46	2169	78%	131452	84313	39%
2019	144	51.6	26.40%	0.37	7.88	95.50%	460.4	1448	76%	124535	64884	34%
2020		74.6			10.5			2198			80595	
2021		74			11.36			2501			82322	
2022		87.8			12.27			2549			89262	
Mean	174	72	29.20%	0.45	5.77	94.50%	615	1034	58%	124938	4.5E+07	30%
Mean Post 2009	179	74	28.60%	0.44	9.85	95.30%	576	2020	76%	130170	74353	38%

SI Table 4

Selenium and Nitrate treatment, locations, timing, and volumetric capacity as presented in the 2022 Teck Implementation Plan Adjustment.[21] Pilot phase start dates sourced from mass removal data provided by Teck.[6] Note, from pilot phase start to the operational date, treatment facilities did not operate continuously and additional treatment pilot projects occurred before 2015.

Water Treatment Facility	Facility Type	Pilot Phase Start	Operational Date	Hydraulic Capacity (m^3/day)
Line Creek Operation WLC Phase I	Active Water Treatment	October 25, 2015	December 31, 2018	6,000
Line Creek Operation WLC Phase II	Active Water Treatment	NA	January 1, 2020	1,500
Elkview Operation SRF Phase I	Saturated Rock Fill Treatment	January 1, 2018	September 1, 2021	20,000
Fording River Operation AWTF (FRO-South)	Active Water Treatment	December 22, 2021	September 1, 2022	20,000

SI Figure 2

Map illustrating Canadian Water Survey of Canada discharge monitoring stations (halo symbols) and water quality sampling locations (triangles) for both the Elk (orange) and Kootenay (blue) Rivers.

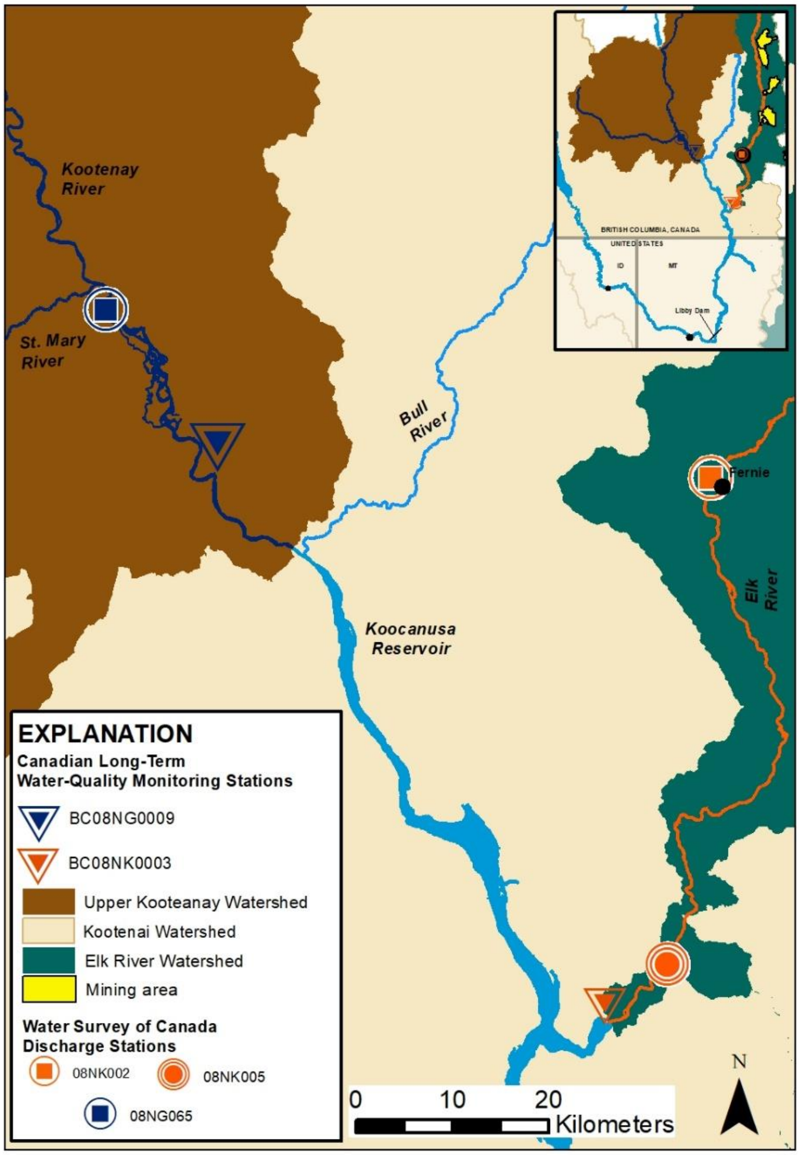

SI Figure 3

Mean daily hodographs for the Elk River at Hwy 93 (top) and Kootenay River at Fenwick (bottom)[22], discharge in m^3/sec. Two time periods are shown one from 1977-2000 and from 2000-2022.

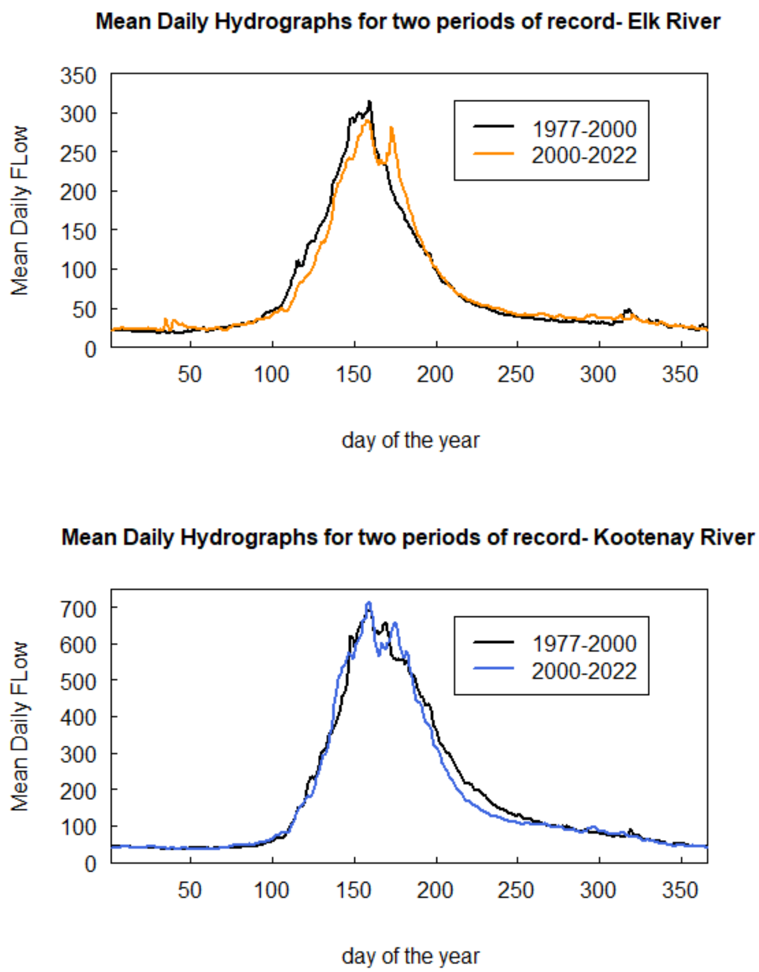

SI Figure 4

Box plot illustrating the range of discharge on days with water quality sampling vs those without water quality samples for Se in the Elk River at Highway 93.

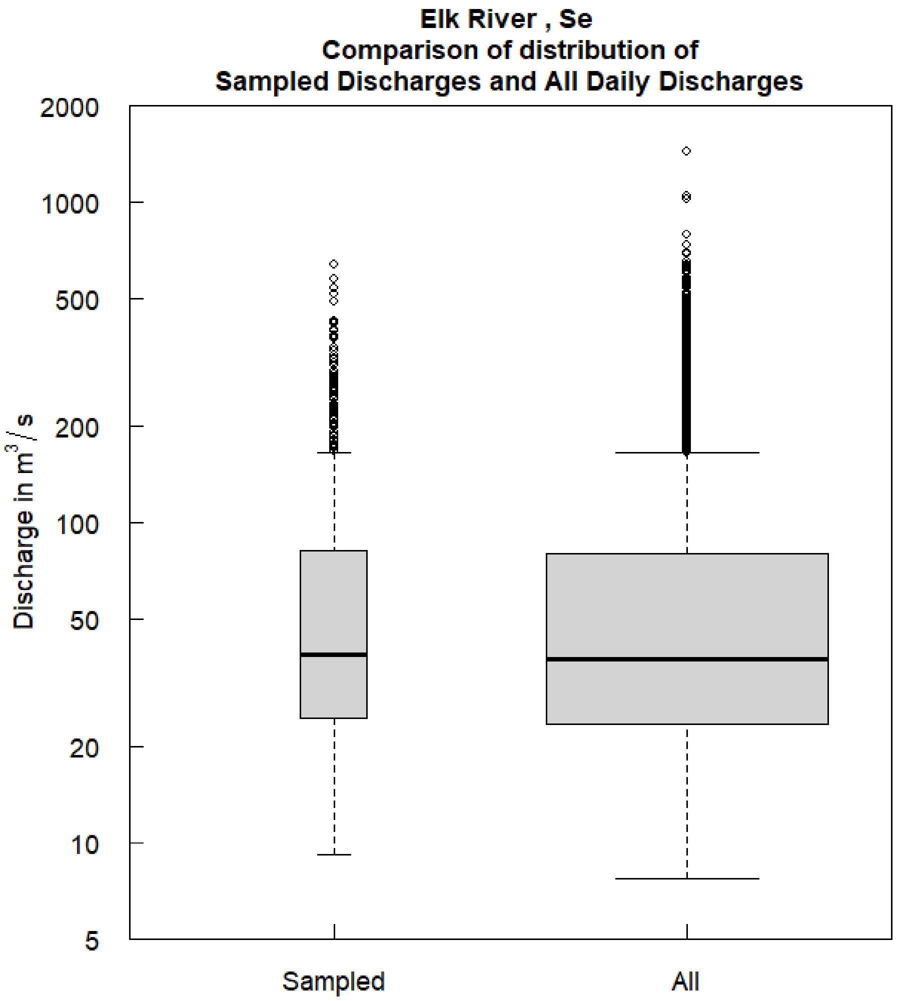

SI Figure 5

Elk River at Highway 93, flow record from 1979-2021. [6] Plots showing 8 different statistical flow metrics, with the Mann-Kendall trend test and corresponding Theil-Sen slope estimate to evaluate flow stationarity. The Theil-Sen slope provides an estimate of the direction and magnitude of the Mann-Kendall trend. [23]

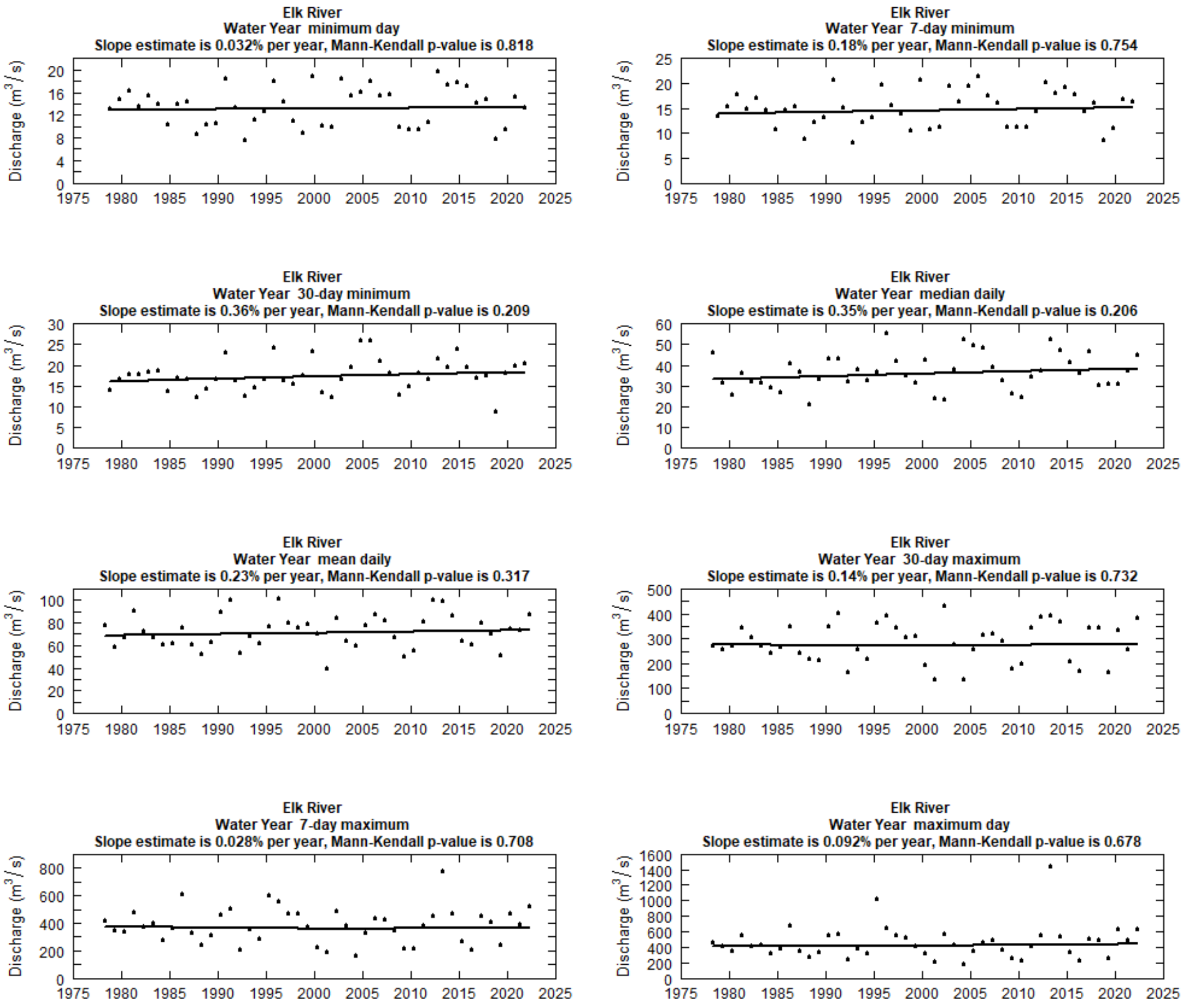

SI Figure 6

Kootenay River at Fenwick, flow record from 1979-2019.[6] Plots showing 8 different statistical flow metrics, and the Mann-Kendall trend test and corresponding Theil-Sen slope estimate to evaluate flow stationarity. The Theil-Sen slope provides an estimate of the direction and magnitude of the Mann-Kendall trend.[23]

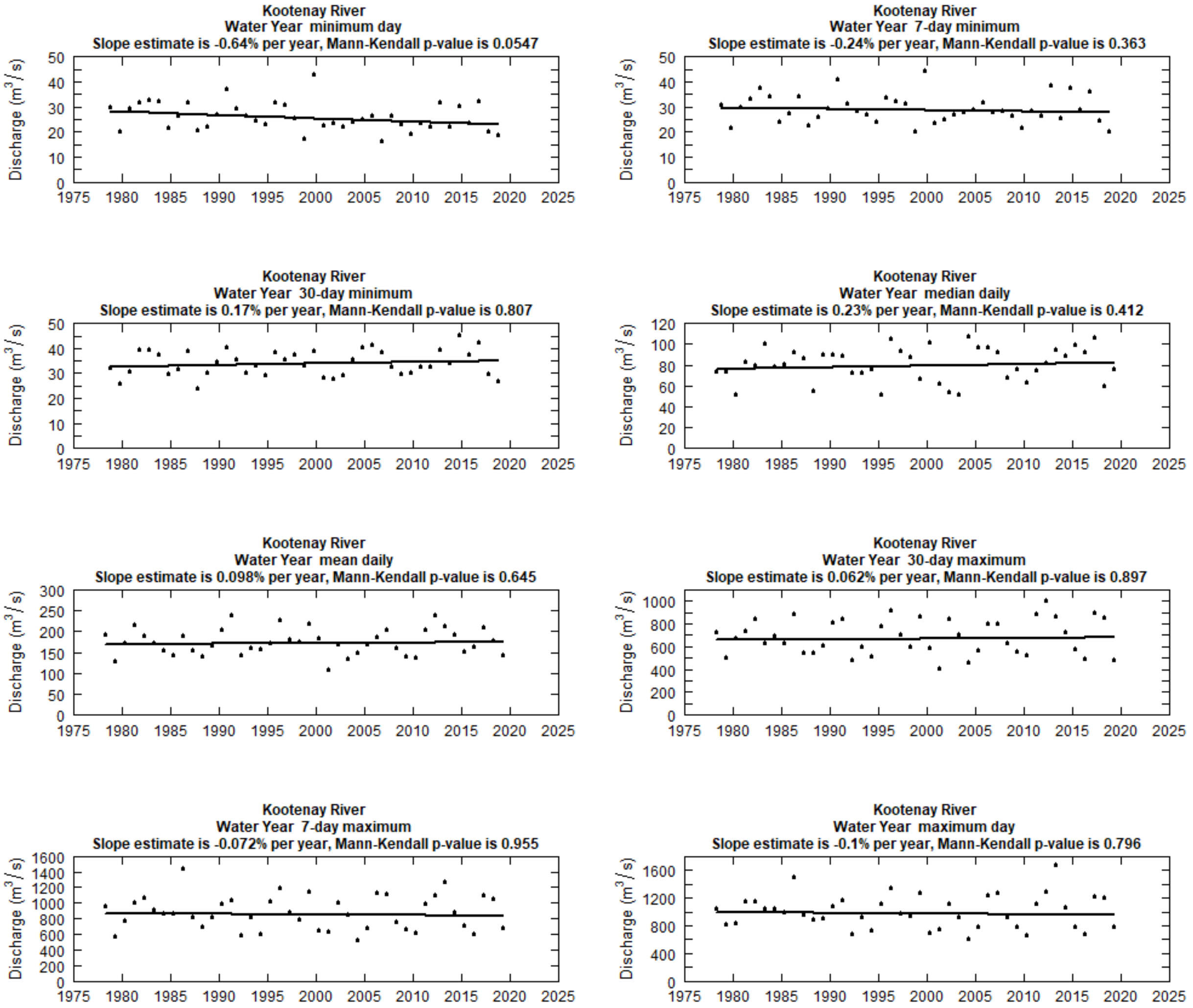

SI Figure 7

Streamflow trends for the Elk River at Highway 93 (top two panels) and the Kootenay River at Fenwick (bottom two panels). Left panels are Quantile-Kendall plots for the water year. Right are Quantile-Kendall plots for the low flow period (February-October). Quantile-Kendall plots are visualizations of trends in slope for ranked discharge, where each point is a trend slope for a given order statistic, the lowest daily discharge is on the left and the highest is on the right. [24]

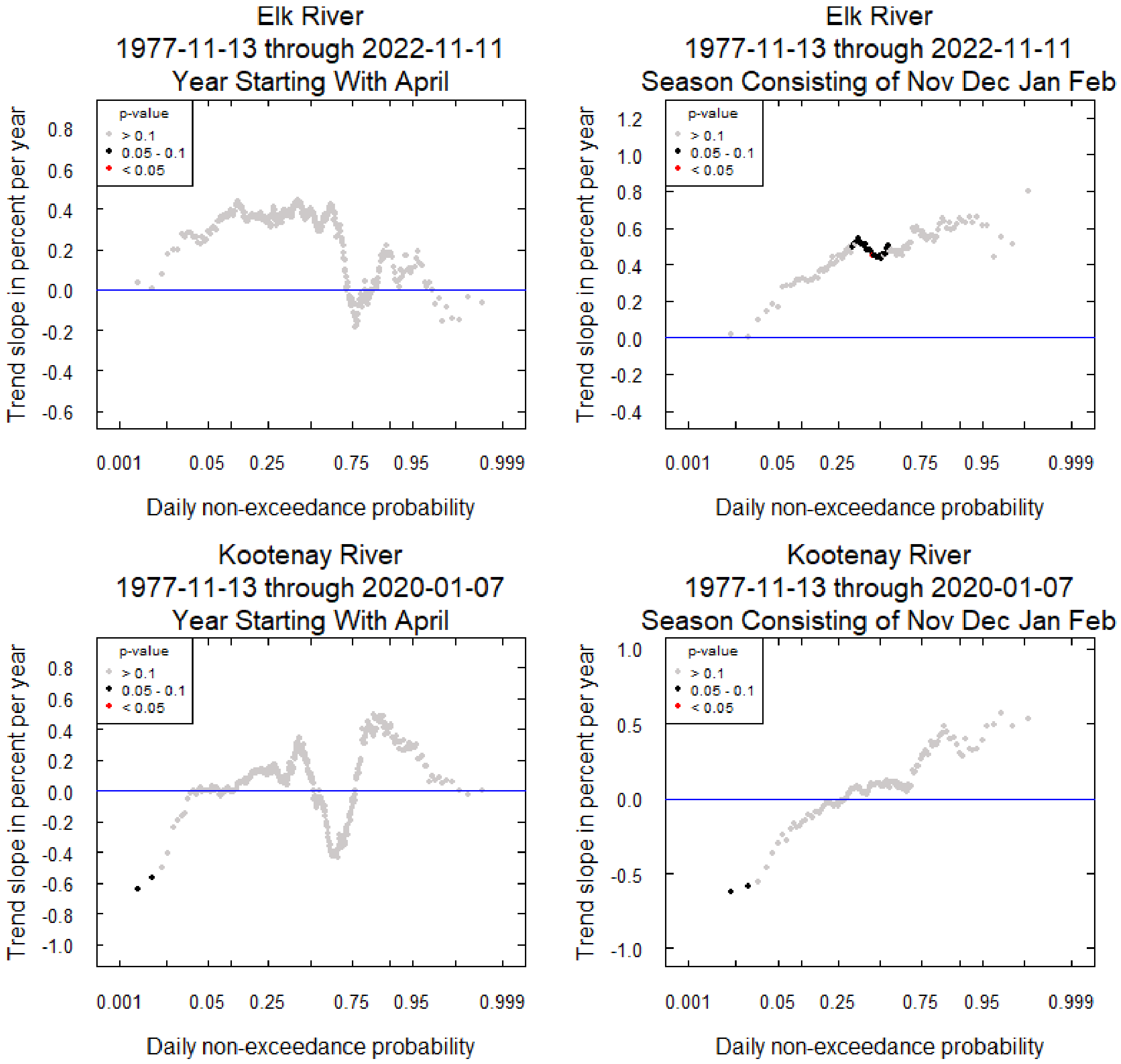

SI Figure 8

Plot illustrating the difference between observed daily loads on the days when samples were collected vs modeled daily load on the same day for Se in the Elk River at Highway 93. Open circles are generated values that represent censored (below detection limit) values. Equivalent R-squared value for this model (error in the estimates of log(Flux)) is 0.852. 20 of the 841 samples were below the detection limit. Models are available in Lange and Storb, 2023.[6]

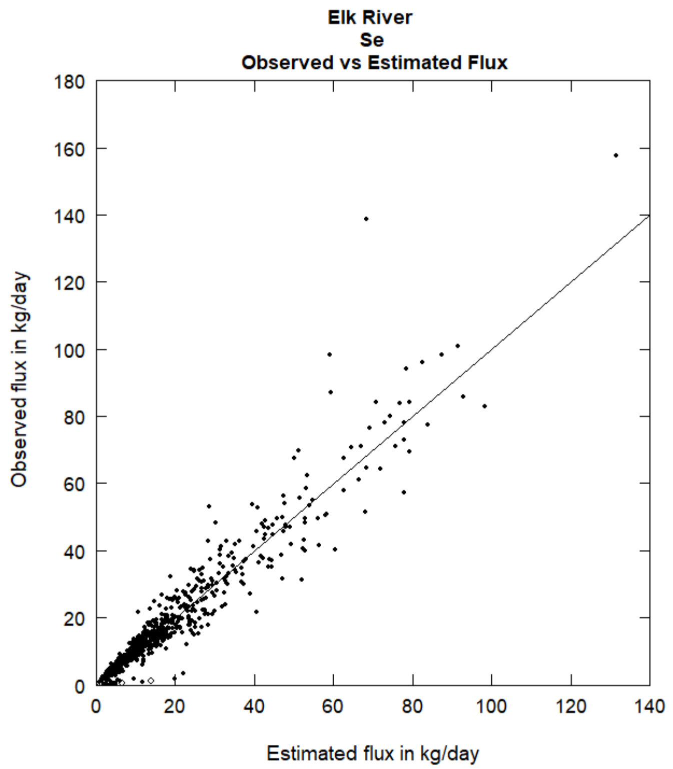

SI Figure 9

Two plots illustrating the difference between WRTDS and WRTDS_kalman models for Se in the Elk River at Highway 93. Models are available in Lange and Storb, 2023.[6]

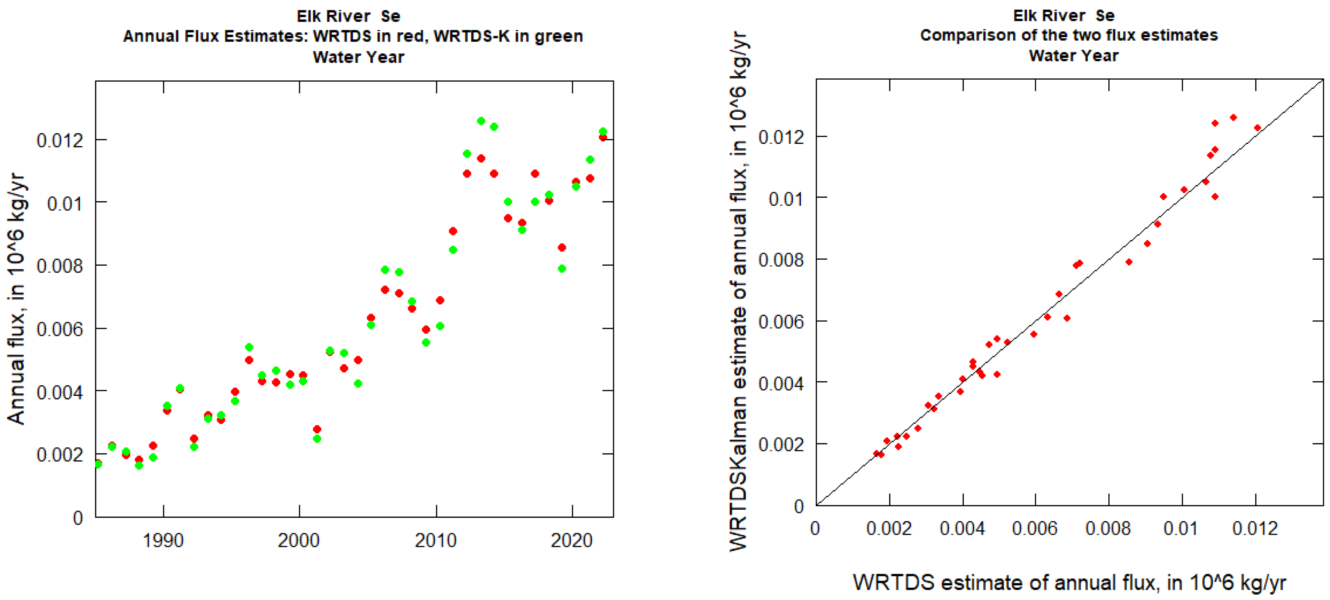

SI Figure 10

Weighted Regression based on Time Discharge and Season (WRTDS) modeled 3-D surfaces for Se, NO_3^-, SO_4^{2-} for the Elk River at Highway 93 and the Kootenay River at Fenwick. Plots illustrate and quantify the relationship between concentration and discharge over time for each WRTDS model. Left plots are the three solutes for the Elk River at Highway 93 and Right plots are the three solutes for the Kootenay River at Fenwick. Top row of plots is Se, middle is NO_3^-, bottom row is SO_4^{2-}. The color ramp represents concentration of the respective constituent in mg/l. Models are available in Lange and Storb, 2023.[6]

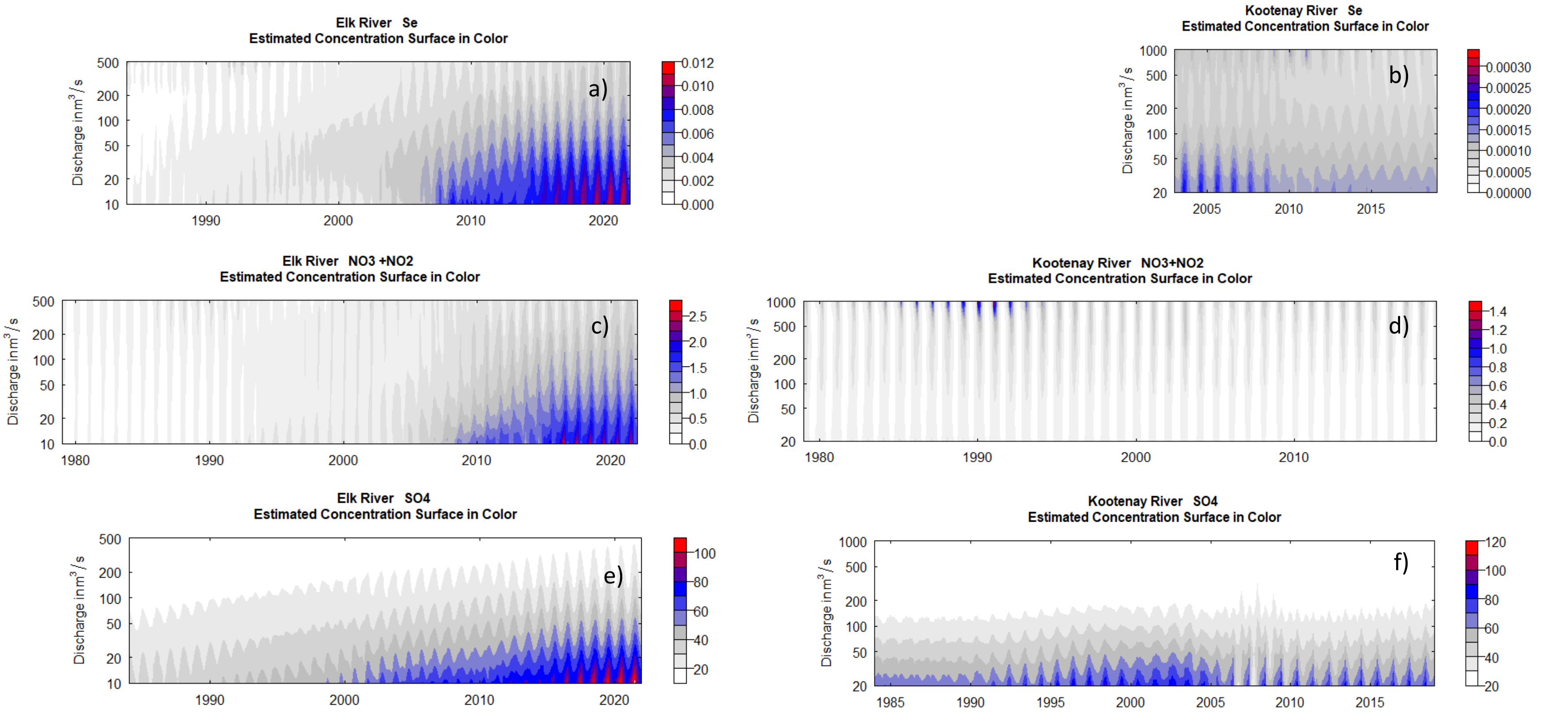

SI Figure 11

Modeled concentration vs discharge relationships. Top row (a-c) is the Kootenay River at Fenwick, the bottom row is the Elk River at Highway 93 (d-e). Each column is a solute, from left to right (Se; a & d, NO_3^-: b & e, SO_4^{2-}; c & f). Each line is a different date. These plots are focused on high flow times of the year (June 13) over time. Low flow times (ex. January 1) exhibit similar patterns but are not shown. Models are available in Lange and Storb, 2023.[6]

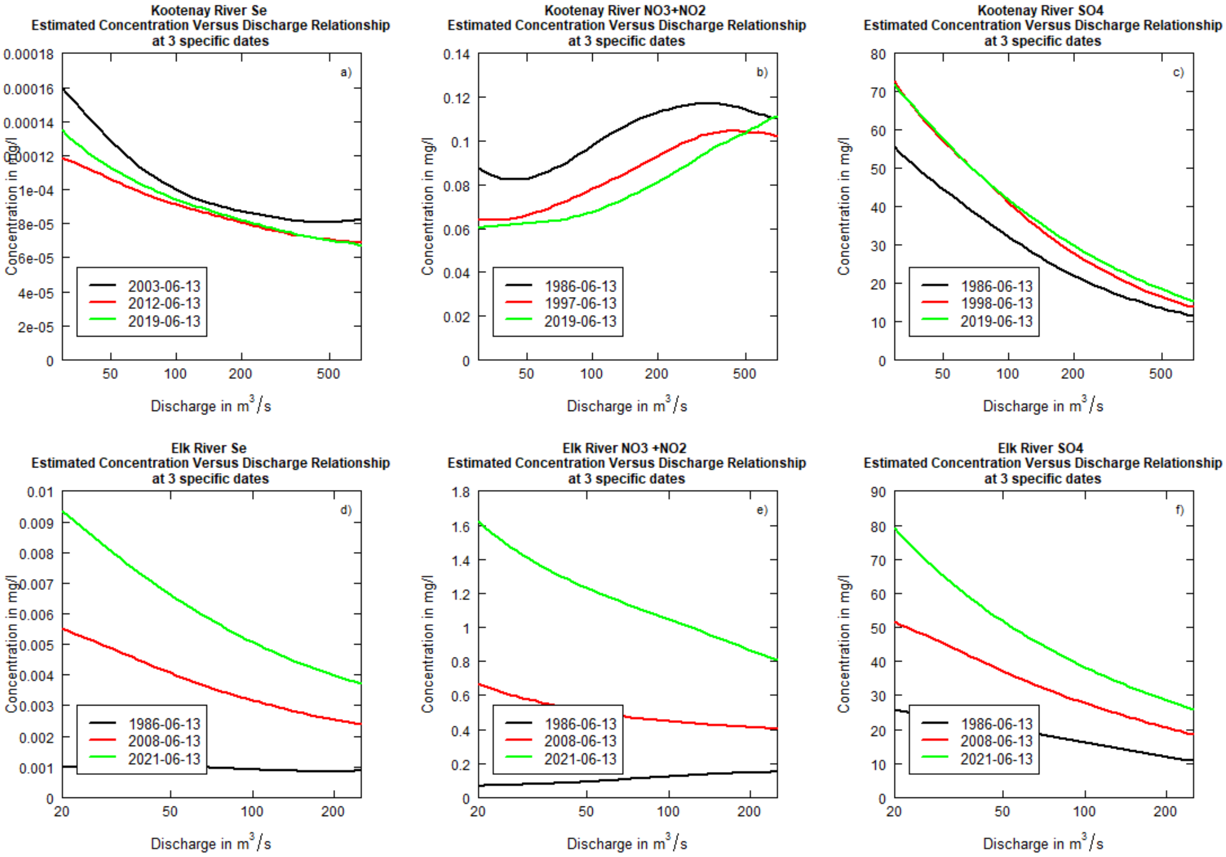

SI Figure 12

Changes in concentration over time at different discharges and times of the year for the Elk River at Highway 93. The left column (a-c-e.) is high discharge time of year (June 13) and the right column (b-d-f.) is a low discharge time of year (Jan 1). Discharge lines roughly represent the 5th, 50th, and 95th percentiles for flow duration curves for 60 days around (30 days before and after) each date. Models are available in Lange and Storb, 2023.[6]

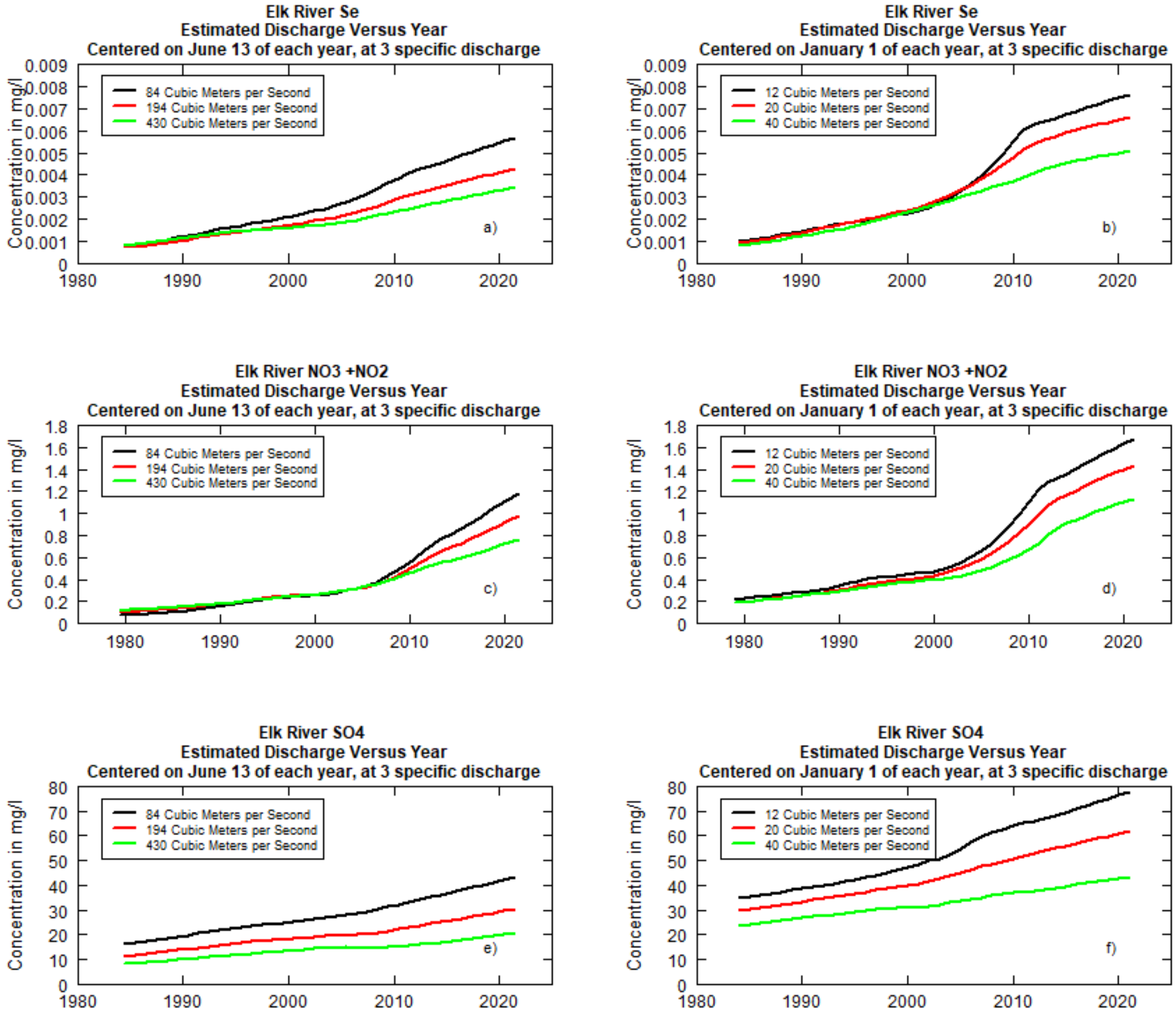

SI Figure 13

Additional perspective on Elk River Mine water treatment. a.) Estimated percentage reduction in concentration due to treatment. Reductions range from 0% to 40%. b.) Boxplots of estimated daily concentrations of Se during the high discharge months of May, June, and July. First box is for water years 2006-2015, second is 2016 – 2022, and the third is also for 2016-2022 but simulated as if there had been no treatment upstream. [the medians of the three boxes shown are 0.0032, 0.0042, 0.0045]. c.) Boxplots of estimated daily concentrations of Se during the low discharge months of August through April. First box is for water years 2006-2015, second is 2016 – 2022, and the third is also for 2016-2022 but simulated as if there had been no treatment upstream [the medians of the three boxes shown are 0.0049, 0.0062, 0.0075].

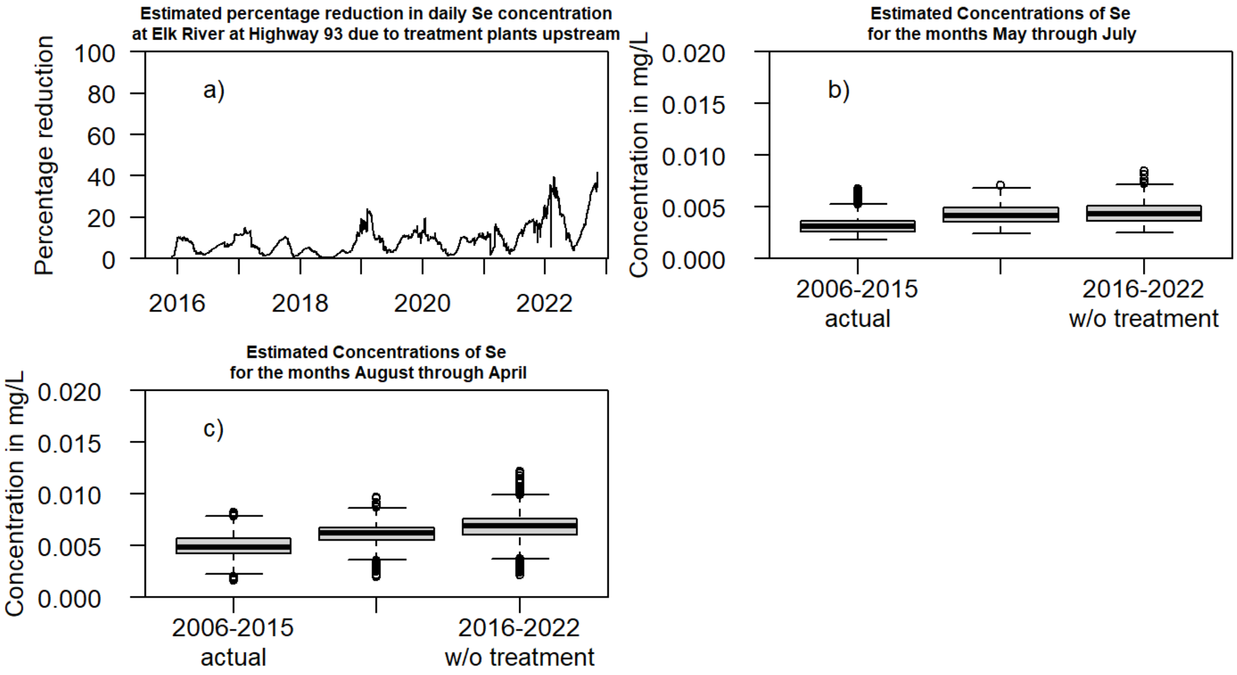

Supporting Information References

(1) Copernicus Sentinel data processed by European Space Agency. (accessed January 18, 2023).

(2) Gorelick, N.; Hancher, M.; Dixon, M.; Ilyushchenko, S.; Thau, D.; Moore, R. Google Earth Engine: Planetary-scale geospatial analysis for everyone. *Remote Sensing of Environment* **2017**, *202*, 18-27. DOI: 10.1016/j.rse.2017.06.031.

(3) Rodriguez-Galiano, V. F.; Ghimire, B.; Rogan, J.; Chica-Olmo, M.; Rigol-Sanchez, J. P. An assessment of the effectiveness of a random forest classifier for land-cover classification. *ISPRS Journal of Photogrammetry and Remote Sensing* **2012**, *67*, 93-104. DOI: 10.1016/j.isprsjprs.2011.11.002.

(4) *ArcGIS Desktop*; Redlands, CA, 2013. (accessed June 22, 2023).

(5) British Columbia Ministry of Forests Lands Natural Resource Operations and Rural Development. *BC Water Tool - Watershed*. 2023. https://kwt.bcwatertool.ca/watershed (accessed February 9, 2023).

(6) Lange, D. A.; Storb, M. B. *Input files and WRTDS model output for the two major tributaries of Lake Koocanusa*; 2023. (accessed June 2023). DOI: 10.5066/P9JP8VGL.

(7) Granato, G. E. *Computer programs for obtaining and analyzing daily mean streamflow data from the U.S. Geological Survey National Water Information System web site*; 2009. DOI: 10.3133/ofr20081362.

(8) Medalie, L. *Effects of urban best management practices on streamflow and phosphorus and suspended-sediment transport on Englesby Brook in Burlington, Vermont, 2000-2010*; Reston, VA, 2012. DOI: 10.3133/sir20125103.

(9) Lorenz, D. L. *smwrBase—An R package for managing hydrologic data, version 1.1.1*; Reston, VA, 2015. (accessed May 8, 2023). DOI: 10.3133/ofr20151202.

(10) Katay, F. *Exploration and mining in the Kootenay-Boundary Region, British Columbia. In: Exploration and Mining in British Columbia, 2014*; Information Circular 2015-2; British Columbia Ministry of Energy and Mines, British Columbia Geological Survey, 2015. https://cmscontent.nrs.gov.bc.ca/geoscience/publicationcatalogue/InformationCircular/BCGS_IC2016-01-03.pdf (accessed February 9, 2023).

(11) Goodarzi, F.; Grieve, D. A.; Sanei, H.; Gentzis, T.; Goodarzi, N. N. Geochemistry of coals from the Elk Valley coalfield, British Columbia, Canada. *International Journal of Coal Geology* **2009**, *77* (3), 246-259. DOI: 10.1016/j.coal.2008.08.010.

(12) Wellen, C.; Shatilla, N. J.; Carey, S. K. The influence of mining on hydrology and solute transport in the Elk Valley, British Columbia, Canada. *Environmental Research Letters* **2018**, *13* (7), 074012. DOI: 10.1088/1748-9326/aaca9d.

(13) British Columbia Forest Analysis and Inventory Branch. *Reconnaissance karst potential mapping*; 2023. https://catalogue.data.gov.bc.ca/dataset/reconnaissance-karst-potential-mapping (accessed May 10, 2023).

(14) Lussier, C.; Veiga, V.; Baldwin, S. The geochemistry of selenium associated with coal waste in the Elk River Valley, Canada. *Environmental Geology* **2003**, *44* (8), 905-913. DOI: 10.1007/s00254-003-0833-y.

(15) Teck Resources Limited. *Sullivan Mine*. 2023. https://www.teck.com/operations/canada/legacy/sullivan-mine/ (accessed June 6, 2023).

(16) Hirsch, R. M.; Moyer, D. L.; Archfield, S. A. Weighted Regressions on Time, Discharge, and Season (WRTDS), with an application to Chesapeake Bay River inputs. *Journal of the American Water Resources Association* **2010**, *46* (5), 857-880. DOI: 10.1111/j.1752-1688.2010.00482.x.

(17) Hirsch, R. M.; Archfield, S. A.; De Cicco, L. A. A bootstrap method for estimating uncertainty of water quality trends. *Environmental Modelling & Software* **2015**, *73*, 148-166. DOI: 10.1016/j.envsoft.2015.07.017.

(18) Hirsch, R. M.; De Cicco, L. A. *User guide to Exploration and Graphics for RivEr Trends (EGRET) and dataRetrieval: R packages for hydrologic data: U.S. Geological Survey Techniques and Methods 4-A10*; Reston, VA, 2015. (accessed February 9, 2023). DOI: 10.3133/tm4A10.

(19) Zhang, Q.; Hirsch, R. M. River water-quality concentration and flux estimation can be improved by accounting for serial correlation through an autoregressive model. *Water Resources Research* **2019**, *55* (11), 9705-9723. DOI: 10.1029/2019WR025338.

(20) Lee, C. J.; Hirsch, R. M.; Crawford, C. G. *An evaluation of methods for computing annual water-quality loads: U.S. Geological Survey Scientific Investigations Report 2019-5084*; Reston, VA, 2019. (accessed February 9, 2023). DOI: 10.3133/sir20195084.

(21) Teck Resources Limited. *Elk Valley water quality plan: 2022 implementation plan adjustment*; 2022. https://www.teck.com/media/EVWQP_2022_ImplementationPlanAdjustment_Main_Report.pdf (accessed January 9, 2023).

(22) British Columbia Ministry of Forests Lands Natural Resource Operations and Rural Development. *B.C. Water Tool - Streamflow & Water Levels*. 2023. https://kwt.bcwatertool.ca/streamflow (accessed June 6, 2023).

(23) Helsel, D. R.; Hirsch, R. M.; Ryberg, K. R.; Archfield, S. A.; Gilroy, E. J. *Statistical methods in water resources*; Reston, VA, 2020. (accessed February 13, 2023). DOI: 10.3133/tm4A3.

(24) U.S. Geological Survey. *Daily Streamflow and Trend Analysis*. 2023. https://doi-usgs.github.io/EGRET/articles/streamflow_trend.html#the-quantile-kendall-plot (accessed February 9, 2023).
